# Slower Learning Rates from Negative Outcomes in Substance Use Disorder over a 1-Year Period and Their Potential Predictive Utility

**DOI:** 10.5334/cpsy.85

**Published:** 2022-06-08

**Authors:** Ryan Smith, Samuel Taylor, Jennifer L. Stewart, Salvador M. Guinjoan, Maria Ironside, Namik Kirlic, Hamed Ekhtiari, Evan J. White, Haixia Zheng, Rayus Kuplicki, Tulsa 1000 Investigators, Martin P. Paulus

**Affiliations:** 1 Laureate Institute for Brain Research, Tulsa, OK, USA; 2 Department of Community Medicine, University of Tulsa, Tulsa, OK USA

**Keywords:** Substance Use Disorders, Computational Modeling, Active Inference, Learning Rate, Explore-Exploit Dilemma, Directed Exploration

## Abstract

Computational modelling is a promising approach to parse dysfunctional cognitive processes in substance use disorders (SUDs), but it is unclear how much these processes change during the recovery period. We assessed 1-year follow-up data on a sample of treatment-seeking individuals with one or more SUDs (alcohol, cannabis, sedatives, stimulants, hallucinogens, and/or opioids; *N* = 83) that were previously assessed at baseline within a prior computational modelling study. Relative to healthy controls (HCs; *N* = 48), these participants were found at baseline to show altered learning rates and less precise action selection while completing an explore-exploit decision-making task. Here we replicated these analyses when these individuals returned and re-performed the task 1 year later to assess the stability of baseline differences. We also examined whether baseline modelling measures could predict symptoms at follow-up. Bayesian and frequentist analyses indicated that: (a) group differences in learning rates were stable over time (posterior probability = 1); and (b) intra-class correlations (ICCs) between model parameters at baseline and follow-up were significant and ranged from small to moderate (.25 ≤ ICCs ≤ .54). Exploratory analyses also suggested that learning rates and/or information-seeking values at baseline were associated with substance use severity at 1-year follow-up in stimulant and opioid users (.36 ≤ *r*s ≤ .43). These findings suggest that learning dysfunctions are moderately stable during recovery and could correspond to trait-like vulnerability factors. In addition, computational measures at baseline had some predictive value for changes in substance use severity over time and could be clinically informative.

## 1. Introduction

Substance use disorders (SUDs) are among the most common, costly, and burdensome psychiatric conditions (NIMH, 2007; Suzuki & Kober, 2018). Despite considerable research to date (Everitt & Robbins, 2016; Valyan, Ekhtiari, Smith, & Paulus, 2020), understanding of the cognitive and neurobiological underpinnings of these conditions remains incomplete, with limited ability to inform treatment or predict symptom change over time. Computational modelling represents a promising approach for further elucidating the neural and cognitive mechanisms underlying SUDs. This approach can account for maladaptive perceptual, learning, and decision-making processes, as well as generate quantitative hypotheses at multiple levels of description. Several computational modelling and neuroimaging studies over the last two decades have found evidence that compulsive behavior patterns seen in SUDs are associated with a shift from so-called ‘model-based’ (goal-directed) to ‘model-free’ (habitual) control (Donamayor, Strelchuk, Baek, Banca, & Voon, 2018; Everitt & Robbins, 2005, 2016; Obst et al., 2018; Reiter et al., 2016; Sebold et al., 2014; Sjoerds et al., 2013; Voon et al., 2015). Other modelling studies have also reported evidence of altered interoception (Smith, Kuplicki, et al., 2020) and altered approach-avoidance processes in SUDs (Smith, Kirlic, Stewart, Touthang, Kuplicki, Khalsa, et al., 2021; Smith, Kirlic, Stewart, Touthang, Kuplicki, McDermott, et al., 2021). These and other types of maladaptive behavior patterns have been linked to relapse as well as several other negative long-term outcomes (Passetti, Clark, Mehta, Joyce, & King, 2008; Verdejo-Garcia, Chong, Stout, Yucel, & London, 2018). As part of the broader field of computational psychiatry (Huys, Maia, & Frank, 2016), the goal of model-based studies has been to identify and measure differences in the information processing mechanisms that underlie such maladaptive patterns, and to examine if they can aid in assessing symptom severity, guiding treatment decisions, predicting treatment outcomes, and evaluating treatment progress, among others (Smith, Taylor, & Bilek, 2021).

This aim of computational psychiatry to inform personalized medicine approaches – via either treatment prediction or assessment of treatment progress – requires that computational measures provide reliable individual difference estimates over time. That is, measures of computational mechanisms should be consistent over time unless true mechanistic changes have occurred. If changes over time instead reflect random influences, their use as assessment tools will be limited (Nair, Rutledge, & Mason, 2020). To address this, the longitudinal stability of computational measures has been the topic of recent studies, with results ranging from poor to excellent estimates of reliability (Brown, Chen, Gillan, & Price, 2020; Chung et al., 2017; Enkavi et al., 2019; Hedge, Bompas, & Sumner, 2020; Moutoussis et al., 2018; Price, Brown, & Siegle, 2019; Shahar et al., 2019; Smith, Kirlic, Stewart, Touthang, Kuplicki, McDermott, et al., 2021). This highly variable pattern of results suggests that there may be significant measurement error and/or that the cognitive processes engaged during many tasks change with repeated performance (e.g., due to learning). Many commonly used computational tasks are also yet to be assessed for longitudinal stability, or for their ability to track or predict changes over time in clinically relevant variables (e.g., symptom levels, physiological states, etc.). There is thus a need for thorough assessment of the longitudinal reliability of a broader range of task measures within computational psychiatry and for further evaluation of their ability to capture information about states vs. traits.

In a recent paper studying SUDs (Smith, Schwartenbeck, et al., 2020), we used a computational modelling approach to analyze behavior on a commonly used three-armed bandit task (Zhang & Yu, 2013), which is designed to measure the balance between information-seeking and reward-seeking during decision-making under uncertainty (i.e., solving the ‘explore-exploit dilemma’; (Addicott, Pearson, Sweitzer, Barack, & Platt, 2017)). This dataset included healthy controls (HCs; *N* = 54) and a community sample of individuals with one or more SUDs (alcohol, cannabis, sedatives, stimulants, hallucinogens, and/or opioids; *N* = 147). This was part of the Tulsa 1000 (T1000) project (Victor et al., 2018) – a naturalistic longitudinal study recruiting subjects based on the dimensional NIMH Research Domain Criteria framework (Insel et al., 2010). Computational modelling in that prior study provided evidence that, relative to HCs, substance users learned more slowly from losses and more quickly from wins. Substance users also showed less precise (less value-sensitive) decisions, corresponding to a behavioral tendency to change decision strategies despite prior success. While these results suggested a mechanism whereby substance users may continue with maladaptive behavior (under uncertainty) despite negative consequences, the stability of these differences was not addressed. Namely, it was not clear whether these results reflected stable trait vulnerability factors, or were dependent on current psychological states, or whether they may track symptom changes over time.

Participants in the T1000 project were invited to return for a 1-year follow-up visit and asked to complete – among other assessments – the above-mentioned three-armed bandit task. This afforded the opportunity to (1) test the individual- and group-level stability of baseline results over time (i.e., whether/how computational phenotypes may change during the recovery process), and (2) examine whether baseline computational measures predict clinical differences at follow-up. This study reports the results of these analyses as a means of examining the clinical utility of this task/model as a potential clinical assessment tool.

## 2. Methods

### 2.1 Participants

Participants represent a subset of those from our original baseline study (Smith, Schwartenbeck, et al., 2020) who agreed to return for a 1-year follow-up visit. In the baseline study, these participants were identified from the exploratory subsample (i.e., first 500 participants) of the T1000 project (Victor et al., 2018), which recruited a community sample of subjects based on the dimensional NIMH Research Domain Criteria framework. The T1000 study included individuals 18–55 years old, screened on the basis of dimensional psychopathology scores: Drug Abuse Screening Test (DAST-10 (Bohn, Babor, & Kranzler, 1991)) score > 3, Patient Health Questionnaire (PHQ-9 (Kroenke, Spitzer, & Williams, 2001)) ≥ 10, and/or Overall Anxiety Severity and Impairment Scale (OASIS (Norman, Hami Cissell, Means-Christensen, & Stein, 2006)) ≥ 8. HCs did not have psychiatric diagnoses or show elevated symptoms. Participants were excluded if they: (a) tested positive for drugs of abuse via urine screen, (b) met criteria for psychotic, bipolar, or obsessive-compulsive disorders, or (c) reported history of moderate-to-severe traumatic brain injury, neurological disorders, severe or unstable medical conditions, active suicidal intent or plan, or change in medication dose within 6 weeks. See (Victor et al., 2018) for a more complete description of inclusion/exclusion criteria. The study was approved by the Western Institutional Review Board. All participants provided written informed consent prior to completion of the study protocol, in accordance with the Declaration of Helsinki, and were compensated for participation. ClinicalTrials.gov identifier: #NCT02450240.

After baseline screening, participants were grouped based on DSM-IV-TR or DSM-5 diagnosis using the Mini International Neuropsychiatric Inventory (MINI version 6.0 or 7.0) (D. Sheehan et al., 2015; D. V. Sheehan & Lecrubier, 2010; D. V. Sheehan et al., 1998). In our baseline study, we focused on treatment-seeking individuals with SUDs (*N* = 147; including alcohol, cannabis, sedatives, stimulants, hallucinogens, and/or opioid use disorder) with or without comorbid depression and anxiety disorders. These individuals were compared to 54 HCs with no mental health diagnoses. Most substance users were currently enrolled in a residential facility or maintenance outpatient program after completion of more intensive treatments (mean days abstinent = 92; SD = 56). Due to a difference between HCs and SUDs in scores on the Wide Range Achievement Test (WRAT) – a commonly used measure of premorbid IQ (Johnstone, Callahan, Kapila, & Bouman, 1996) – our prior study also confirmed results in a subsample propensity matched on this measure (as well as on age and sex). This included 51 HCs and 49 SUDs. Of the participants who were invited to return for the 1-year follow-up, 48 HCs and 83 substance users agreed to participate (45 HCs and 25 SUDs in the propensity matched subsample). Table 1 lists group demographics and clinical measures for both the baseline and follow-up samples by group (only including those that returned for follow-up). Table 2 also lists diagnosis frequency for specific SUDs and anxiety/depression for baseline and follow-up (including all participants, showing that diagnostic composition did not change with dropout).

**Table 1:** Descriptive Statistics (Means and Standard Deviations) for Demographic and Clinical Measures by Group and Session.


FULL SAMPLE									

	TOTAL	BASELINE		FOLLOW-UP		USABLE DATA (N)	EFFECT OF CLINICAL STATUS	EFFECT OF SESSION	EFFECT OF CLINICAL STATUS/SESSION INTERACTION
	
		HCS	SUDS	HCS	SUDS				
	
	131	48	83	48	83				

**Age**	34.17 (10.18)	32.19 (11.19)	35.32 (9.46)	N/A	N/A	HC: 48 SUD +: 83 Total: 131	t(129) = –1.7 *p* = 0.09 d = –0.3	N/A	N/A

**Sex (Male)**	114 (44%)	22 (46%)	35 (42%)	N/A	N/A	HC: 48 SUD +: 83 Total: 131	χ^2^(1) = 0.17 *p* = 0.68	N/A	N/A

**DAST**	3.48 (3.72)	0.12 (0.39)	7.55 (2.17)	0.45 (0.55)	2.72 (3.01)	HC: 38 SUD +: 81 Total: 119	***F*(1, 117) = 193.62** ***p* < 0.001** **η^2^ = 0.62**	***F*(1, 117) = 177.72** ***p* < 0.001** **η^2^ = 0.6**	***F*(1, 117) = 102.46** ***p* < 0.001** **η^2^ = 0.47**

**PHQ**	3.67 (5.1)	0.88 (1.33)	6.84 (6.11)	1.08 (1.81)	3.35 (4.69)	HC: 38 SUD +: 80 Total: 118	***F*(1, 116) = 30.51** ***p* < 0.001** **η^2^ = 0.21**	***F*(1, 116) = 25.15** ***p* < 0.001** **η^2^ = 0.18**	***F*(1, 116) = 16.44** ***p* < 0.001** **η^2^ = 0.12**

**OASIS**	3.7 (4.35)	1.48 (2.01)	5.99 (4.74)	1.39 (2.27)	3.74 (4.49)	HC: 38 SUD +: 81 Total: 119	***F*(1, 117) = 27.47** ***p* < 0.001** **η^2^ = 0.19**	***F*(1, 117) = 177.72** ***p* < 0.001** **η^2^ = 0.6**	***F*(1, 117) = 8.82** ***p* = 0.004** **η^2^ = 0.07**

**WRAT**	60.42 (6.26)	63.78 (4.61)	58.45 (6.31)	N/A	N/A	HC: 45 SUD +: 77 Total: 122	***t*(120) = 4.94** ***p* < 0.001** **d = 0.9**	N/A	N/A

Regular Nicotine Smoker*	68 (26%)	7 (15%)	27 (33%)	N/A	N/A	HC: 46 SUD +: 47 Total: 93	**χ^2^(1) = 17.87** ***p* < 0.001**	N/A	N/A

**PROPENSITY-MATCHED**									

	**TOTAL**	**BASELINE**		**FOLLOW-UP**		**USABLE DATA (N)**	**EFFECT OF CLINICAL STATUS**	**EFFECT OF SESSION**	**EFFECT OF CLINICAL STATUS/SESSION INTERACTION**
	
		**HCS**	**SUDS**	**HCS**	**SUDS**				
	
	**70**	**45**	**25**	**45**	**25**				

**Age**	32.4 (10.37)	32.27 (11.25)	32.65 (8.91)	N/A	N/A	HC: 45 SUD +: 25 Total: 70	t(68) = –0.14 *p* = 0.89 d = –0.04	N/A	N/A

**Sex (Male)**	68 (49%)	21 (47%)	13 (52%)	N/A	N/A	HC: 45 SUD +: 25 Total: 70	χ2(1) = 0.18 *p* = 0.67	N/A	N/A

**DAST**	2.31 (3.4)	0.13 (0.4)	7.6 (2.36)	0.45 (0.55)	3.76 (3.37)	HC: 38 SUD +: 25 Total: 63	***F*(1, 61) = 186.8** ***p* < 0.001** **η^2^ = 0.75**	***F*(1, 61) = 24.9** ***p* < 0.001** **η^2^ = 0.29**	***F*(1, 61) = 60.05** ***p* < 0.001** **η^2^ = 0.5**

**PHQ**	2.74 (4)	0.87 (1.36)	7.68 (5.15)	1.08 (1.81)	3.72 (3.96)	HC: 38 SUD +: 25 Total: 63	***F*(1, 61) = 63.99** ***p* < 0.001** **η^2^ = 0.51**	***F*(1, 61) = 6.59** ***p* = 0.01** **η^2^ = 0.1**	***F*(1, 61) = 16.84** ***p* < 0.001** **η^2^ = 0.22**

**OASIS**	3.01 (3.8)	1.44 (1.99)	6.72 (4.46)	1.39 (2.27)	4.56 (4.33)	HC: 38 SUD +: 25 Total: 63	***F*(1, 61) = 40.52** ***p* < 0.001** **η^2^ = 0.4**	*F*(1, 61) = 2.69 *p* = 0.11 η^2^ = 0.04	***F*(1, 61) = 5.59** ***p* = 0.02** **η^2^ = 0.08**

**WRAT**	63.29 (4.83)	63.78 (4.61)	62.4 (5.22)	N/A	N/A	HC: 45 SUD +: 25 Total: 70	*t*(68) = 1.14 *p* = 0.26 d = 0.28	N/A	N/A

Regular Nicotine Smoker*	32 (23%)	7 (16%)	9 (36%)	N/A	N/A	HC: 44 SUD +: 17 Total: 61	**χ2(1) = 8.69** ***p* = 0.003**	N/A	N/A


* Defined as >3650 lifetime cigarettes. DAST = Drug Abuse Screening Test. PHQ = Patient Health Questionnaire. OASIS = Overall Anxiety Severity and Impairment Scale. WRAT = Wide Range Achievement Test. Significant effects are bolded.

**Table 2:** Lifetime DSM-IV/DSM-5 psychiatric disorders within SUDs.


	FULL DATASET			PROPENSITY-MATCHED		
	
	BASELINE (N = 147)	FOLLOW-UP (N = 83)	ANALYSIS	BASELINE (N = 49)	FOLLOW-UP (N = 25)	ANALYSIS

**Substance Use Disorders**						

**Alcohol**	55 (37%)	30 (36%)	χ^2^(1) = 0.04 *p* = 0.85	20 (41%)	10 (40%)	χ^2^(1) = 0 *p* = 0.95

**Cannabis**	55 (37%)	23 (28%)	χ^2^(1) = 2.23 *p* = 0.14	17 (35%)	5 (20%)	χ^2^(1) = 1.71 *p* = 0.19

**Stimulants**	104 (71%)	61 (73%)	χ^2^(1) = 0.2 *p* = 0.66	35 (71%)	18 (72%)	χ^2^(1) = 0 *p* = 0.96

**Opioids**	56 (38%)	30 (36%)	χ^2^(1) = 0.09 *p* = 0.77	25 (51%)	14 (56%)	χ^2^(1) = 0.16 *p* = 0.68

**Sedatives**	38 (26%)	21 (25%)	χ^2^(1) = 0.01 *p* = 0.93	14 (29%)	9 (36%)	χ^2^(1) = 0.43 *p* = 0.51

**Hallucinogens**	5 (3%)	3 (4%)	χ^2^(1) = 0.01 *p* = 0.93	2 (4%)	2 (8%)	χ*2(1) = 0.5p = 0.48*

**2+ Disorders**	94 (64%)	51 (61%)	χ^2^(1) = 0.14 *p* = 0.71	34 (69%)	18 (72%)	χ^2^(1) = 0.05 *p* = 0.82

**Alcohol Only**	9 (6%)	6 (7%)	χ^2^(1) = 0.11 *p* = 0.74	3 (6%)	2 (8%)	χ^2^(1) = 0.09 *p* = 0.76

**Cannabis Only**	9 (6%)	4 (5%)	χ^2^(1) = 0.17 *p* = 0.68	4 (8%)	2 (8%)	χ^2^(1) = 0 *p* = 0.98

**Stimulants Only**	26 (18%)	17 (20%)	χ^2^(1) = 0.27 *p* = 0.6	6 (12%)	3 (12%)	χ^2^(1) = 0 *p* = 0.98

**Opioids Only**	8 (5%)	5 (6%)	χ^2^(1) = 0.03 *p* = 0.85	2 (4%)	0 (0%)	χ^2^(1) = 1.05 *p* = 0.31

**Sedatives Only**	0 (0%)	0 (0%)	NA	0 (0%)	0 (0%)	NA

**Mood, Anxiety, Stress Disorders**						

**Major Depressive**	78 (53%)	43 (52%)	χ^2^(1) = 0.03 *p* = 0.85	30 (61%)	15 (60%)	χ^2^(1) = 0.01 *p* = 0.92

**Generalized Anxiety**	22 (15%)	14 (17%)	χ^2^(1) = 0.15 *p* = 0.7	9 (18%)	7 (28%)	χ^2^(1) = 0.91 *p* = 0.34

**Social Anxiety**	19 (13%)	11 (13%)	χ^2^(1) = 0.01 *p* = 0.94	8 (16%)	3 (12%)	χ^2^(1) = 0.24 *p* = 0.62

**Panic**	17 (12%)	10 (12%)	χ^2^(1) = 0.01 *p* = 0.91	7 (14%)	3 (12%)	χ^2^(1) = 0.07 *p* = 0.79

**Post-Traumatic Stress**	23 (16%)	14 (17%)	χ^2^(1) = 0.06 *p* = 0.81	10 (20%)	5 (20%)	χ^2^(1) = 0 *p* = 0.97

**2+ Disorders**	46 (31%)	30 (36%)	χ^2^(1) = 0.56 *p* = 0.45	18 (37%)	10 (40%)	χ^2^(1) = 0.08 *p* = 0.78


*Note*: Stimulants = amphetamine, methamphetamine, and/or cocaine.

### 2.2 Procedure

T1000 participants underwent a thorough assessment of demographic, clinical and psychiatric factors. The complete list of assessments and supportive references are provided in (Victor et al., 2018). Here we focus on the same symptom measures assessed in the baseline study (i.e., DAST, PHQ, and OASIS).

**Figure 1:**
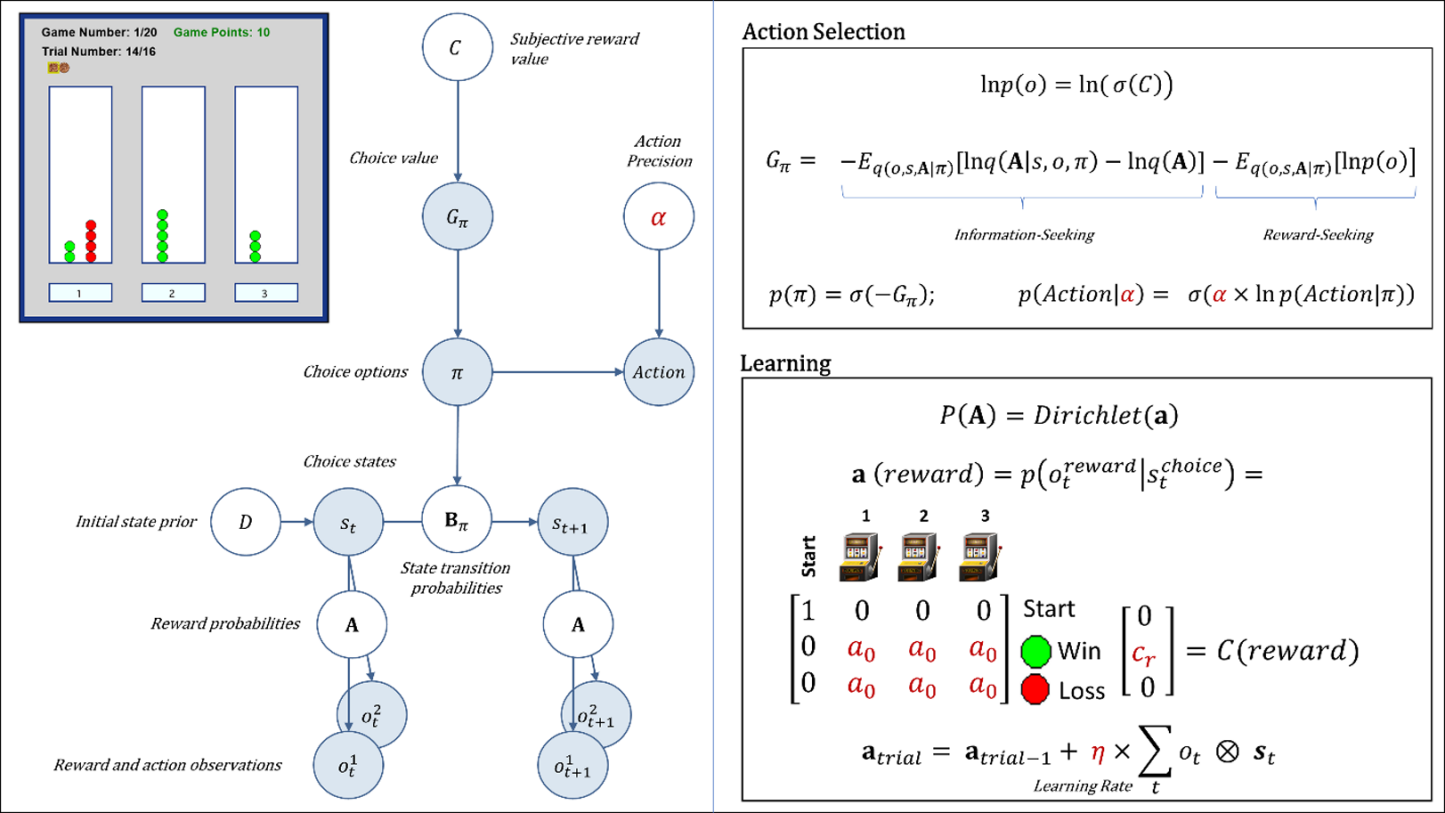
*Upper left*: Illustration of the three-armed bandit task interface. In each of 20 games, participants had 16 opportunities (trials) to choose between one of three options with unknown (but stable) probabilities of winning vs. not winning a point (corresponding to the appearance of a green vs. red circle above the chosen option). Throughout the task, the interface displayed the game number, trial number, total points earned, and history of wins/losses for each choice within the current game (number of green and red circles above each option; see main text for more details). *Left panel*: Graphical depiction of the computational (partially observable Markov decision process) model used with the task (described in the main text). The values of variables in blue circles are inferred on each trial, whereas parameter values in white circles are fixed on each trial. Here, arrows indicate dependencies between variables such that observations \[(o_t^m)\] for each modality *m* (reward and observed choice) at a time *t* depend on choice states (*s_t_*) at time *t*, where these relationships, \[p(o_t^m{\mathrm{|}}{s_t})\], are specified by a matrix **A**. States depend on both previous states and the choice of action policy (*π*), as specified by policy-dependent transition matrices **B***π* that encode *p*(*s_t_*_+1_|*s_t_, π*). States at *t* = 1 have an initial state prior specified by a vector *D*. Here, *D* = [1 0 0 0]*^T^*, such that the participant always started in an undecided ‘start’ state at the beginning of each trial. The probability of selecting an action policy depends on its expected free energy (*Gπ*), which in turn depends on the subjective reward value of making different observations (e.g., a win vs. loss) for the participant (in a vector *C*). These preferences are defined as a participant’s *log-expectations* over observations, \[(o_t^m)\]. As shown in the top-right panel, the values in *C* are passed through a softmax (normalized exponential) function, *σ*(), which transforms them into a proper probability distribution, and then converted into log probabilities. *Top right panel*: Specifies the mathematical form of the dependencies between *C, Gπ, π*, and *a* in action selection. When there is no uncertainty about states (as is true of this task), *Gπ* assigns higher values to actions that are expected to simultaneously maximize information gain and reward. The first term on the right corresponds to expected information gain under approximate posterior beliefs (*q*). Large values for this first term indicate the expectation that beliefs about reward probabilities (**A**) will undergo a large change (i.e., that a lot will be learned about these probabilities) given a choice of policy, due to the states and observations it is expected to generate. The second term on the right motivates reward maximization, where a high reward value corresponds to a precise prior belief over a specific observation, \[p(o_t^m)\]. For example, if the subjective value of a win in *C* were *c_r_* = 4 (see bottom right panel), this would indicate a greater subjective reward (higher prior probability) than *c_r_* = 2. The policy expected to maximize the probability of a win (under the associated beliefs about states, observations, and reward probabilities) is therefore favored. Because the two terms in expected free energy are subtracted, policies associated with high expected reward and high expected information gain will be assigned a lower expected free energy. This formulation entails that information-seeking dominates when reward probabilities are uncertain, while reward-seeking dominates when uncertainty is low. A softmax function, *σ*(), then transforms the negative expected free energies into a probability distribution over policies, such that policies with lower expected free energies are assigned higher probabilities. When actions are subsequently sampled from the posterior distribution over policies, randomness in chosen actions is controlled by an action precision parameter (*a*). *Bottom panel*: After each observation of a win/loss, learning corresponds to updating beliefs in a Dirichlet distribution (**a**) over the likelihood matrix **A** that encodes reward probabilities. Here, columns indicate (from left to right) a starting state (pre-choice) and choices 1, 2, and 3, where the rows (from top to bottom) indicate the pre-choice (no reward) observation, observing reward, or no reward. The value of *a*_0_ – the *insensitivity to information* parameter – is the starting value for beliefs about reward probabilities. These beliefs always start by making up an uninformative (flat) distribution, but higher starting values (e.g., 5 vs. 0.5) effectively down-weight the information-gain term in the expected free energy – leading to an insensitivity to the need for information. The values within **a** (*reward*) are then updated based on the bottom equation, controlled by a learning rate parameter (*η*). For more details regarding the associated mathematics, see the main text and supplemental materials, as well as (Da Costa et al., 2020; K. J. Friston, Lin, et al., 2017; K. J. Friston, Parr, & de Vries, 2017; Smith, Friston, & Whyte, 2022). Estimated model parameters are shown in dark red.

To address our questions about the longitudinal reliability and predictive utility of computational measures gathered at baseline, participants performed the same three-armed bandit task at follow-up (Zhang & Yu, 2013). This task is designed to quantify how individuals switch between an information-seeking and reward-seeking strategy. In each of 20 games, participants had to repeatedly sample from 3 different choice options with unknown (stable) reward probabilities of winning/losing, with the goal of maximizing reward. The optimal strategy is to start by ‘exploring’ (trying all possible options) to gain information about the probability of winning for each option, and then begin ‘exploiting’ after a few trials by repeatedly choosing the option believed to have the highest reward probability. Participants were informed that each game had 16 trials – corresponding to 16 tokens that could be used by pressing one of 3 buttons. The top-left panel of Figure 1 depicts the task interface, which displayed the game number, trial number, and total points earned as participants progressed through the task. After using each token, they earned 1 point if the token turned green or zero points if the token turned red. Each token decision lasted about 2 sec. After the button press, the chosen option became highlighted for 250ms, after which the token turned green or red to reveal the choice outcome. Participants were instructed to find the most rewarding option and maximize the points earned in each game. They were informed that each option had a different (unknown) probability of reward that would not change within a game, but that the probabilities could change at the start of each new game. Reward probabilities were generated from a Beta(2, 2) distribution prior to the start of data collection. Identical reward probabilities were used across participants, with pseudorandomized block order. Participants were paid an additional $5 or $10 based on task performance.

### 2.3 Computational modeling

To model task behavior, we adopted the same partially observable Markov decision process (POMDP) model used at baseline. This approach was motivated by the fact that these models can test for differences in learning rates, random exploration, goal-directed exploration, and sensitivity to information (Schwartenbeck et al., 2019), each of which can contribute to explore/exploit decisions in distinct ways. Estimating the (potentially suboptimal) values of these parameters for each individual can provide insights into the specific decision processes that may promote maladaptive behavior in SUDs (Schwartenbeck et al., 2015). For details about the structure and mathematics of this general class of models, see (Da Costa et al., 2020; Smith, Friston, et al., 2022).

The model is described in full detail in **Supplementary Materials**. Example simulations are also shown in **Supplementary Figure S1**. The model is identical to that used in our previous paper and is outlined in Table 3. The model is also depicted graphically (with associated equations) in Figure 1 and described in detail in the legend. Briefly, the model was defined by (1) the choice states available on each trial in the task, (2) the possible outcomes of those choices (wins/losses), (3) the reward probabilities under each choice state, and (4) the reward value of each possible outcome. Free parameters that influence behavior in the model include: action precision (*a*), reward sensitivity (*c_r_*), learning rate (*η*), and insensitivity to information (*a*_0_). The action precision parameter controls the level of stochasticity in choice. Lower values promote choices that are less consistent with beliefs about reward probabilities. In explore-exploit tasks, this corresponds most closely to the construct of random exploration (i.e., choosing actions more randomly as a means of gathering information in the context of high uncertainty). However, random choices in later trials are less consistent with an exploration-based interpretation. The reward sensitivity parameter reflects how much an individual values observing a win. Importantly, as described in **Supplementary Materials**, decision-making is based on a weighted trade-off between expected reward and expected information gain. This means that lower reward sensitivity values will lead individuals to place more value on information-seeking and lead to greater goal-directed exploration. Learning rates quantify how quickly an individual’s beliefs about reward probabilities change when observing each new win/loss. (i.e., influencing how quickly the value of information decreases over time). Insensitivity to information reflects baseline levels of confidence in beliefs about the probability of wins vs. losses for each choice (i.e., before making any observations). Higher insensitivity also leads to reduced goal-directed exploration, because an individual sees less need to seek information a priori. However, unlike reward sensitivity, the influence of this parameter interacts with learning (i.e., higher values also have the effect of making beliefs about reward probabilities less malleable).

**Table 3:** Computational model description.


MODEL ELEMENT	GENERAL DESCRIPTION	MODEL SPECIFICATION

** \[o_t^m\]**	One vector per modality (*m*) of possible observations. Each vector contains entries corresponding to possible observable stimuli for that category at time *t*.	Possible observations for reward (modality 1): Start Reward No reward Possible observations for choice (modality 2): Start Choice 1 Choice 2 Choice 3

*s_t_*	A vector containing entries corresponding to the probability of each possible state that could be occupied at time *t*.	Possible choice states: Start Choice 1 Choice 2 Choice 3

**A** ** \[p(o_t^m{\mathrm{|}}{s_t})\]**	A matrix encoding the relationship between states and observations (one matrix per outcome modality).	A reward probability matrix: *p*(o*_reward_*|s*_choice_*) An identity matrix for observed choice (entailing that participants had no uncertainty about the choice they made): *p*(o*_choice_*|s*_choice_*)

**a** ** \[p(o_t^m{\mathrm{|}}{s_t})\]**	Dirichlet priors associated with the **A** matrix that specify beliefs about the mapping from states to observations. Learning corresponds to updating the concentration parameters for these priors after each observation, where the magnitude of the updates is controlled by a learning rate parameter *η* (see **Supplementary Materials** and Figure 1).	Each entry for learnable reward probabilities began with a uniform concentration parameter value of magnitude *a*_0_, and was updated after each observed win or loss on the task. The learning rate *η* and *a*_0_ (which can be understood as a measure of sensitivity to new information; see **Supplementary Materials**) were fit to participant behavior.

**B** *p*(*s_t+1_*|*s_t_*,π)	A set of matrices encoding the probability of transitioning from one state to another given the choice of policy (π). Here policies simply include the choice of each bandit.	Transition probabilities were deterministic mappings based on a participant’s choices such that, for example, *p*(*s*_choice 1_|*s_start_,π_option 1_*) = 1, and 0 for all other transitions, and so forth for the other possible choices.

*C* *p*(*o_t_*)	One vector per observation modality (per time point) encoding the preference (subjective reward value) of each possible observation within that modality. This vector is passed through a softmax function and then log-transformed.	The value of observing a win was a model parameter *c_r_* reflecting subjective reward value (reward sensitivity); the value of all other observations was set to 0. The value of *c_r_* was fit to participant behavior. Crucially, higher *c_r_* values have the effect of reducing goal-directed exploration, as the probability of each choice (based on expected free energy *Gπ*) becomes more driven by reward than by information-seeking (see **Supplementary Materials** and Figure 1).

*D* *p*(*s_t_*_=1_)	A vector encoding prior probabilities over states.	This encoded a probability of 1 that the participant began in the start state.

π	A vector encoding the probability of selecting each allowable policy (one entry per policy). The value of each policy is determined by its expected free energy (*Gπ*), which depends on a combination of expected reward and expected information gain. Actions at each time point are chosen based on sampling from the distribution over policies, *π* = *σ* (–*G*); the determinacy of action selection is modulated by an inverse temperature or action precision parameter α (see **Supplementary Materials** and Figure 1).	This included 3 allowable policies, corresponding to the choice of transitioning to each of the three choice states. The action precision parameter α was fit to participant behavior.


Estimating these parameters (*a, c, η, a*_0_) for each individual therefore affords investigation of the mechanisms that can lead to maladaptive choice under uncertainty on an individual basis (Schwartenbeck et al., 2015). Model simulations were run using standard routines available in SPM12 academic software (http://www.fil.ion.ucl.ac.uk/spm/; see software note). As with our prior study, we estimated 10 different nested models, illustrated in Table 4, each with different combinations of possible parameters. Bayesian model comparison was then performed to determine the best model (based on (Rigoux, Stephan, Friston, & Daunizeau, 2014; Stephan, Penny, Daunizeau, Moran, & Friston, 2009)). Variational Bayes (variational Laplace; (K. Friston, Mattout, Trujillo-Barreto, Ashburner, & Penny, 2007)) was used to estimate parameter values that maximized the likelihood of each participant’s responses, as described in (Schwartenbeck & Friston, 2016). After establishing the winning model, we confirmed parameter recoverability by simulating behavior under the range of parameter values observed in participants (i.e., using the same combinations of posterior parameter values inferred from the behavioral data in each subject). We then ran the estimation routine on this behavior and examined correlations between the generative and estimated parameters. We also performed additional diagnostic checks (described in detail within **Supplementary Materials**) to assess model identifiability within Bayesian model comparison and to confirm that parameter estimates in the winning model were not strongly dependent on choice of prior means within variational Bayes.

**Table 4:** Nested models.


PARAMETER:	a (ACTION PRECISION)	*c_r_* (REWARD SENSITIVITY)	*η* (LEARNING RATE)	*a*_0_ (INSENSITIVITY TO INFORMATION)

**Default value if not estimated**	4	(always estimated)	(removed from model)	0.25

Prior means during estimation*	4	4	0.5	0.25

**Model 1**	**Y**	**Y**	N	N

**Model 2**	**Y**	**Y**	**Y**	N

**Model 3**	**Y**	**Y**	**Y**	**Y**

**Model 4**	N	**Y**	**Y**	**Y**

**Model 5**	N	**Y**	**Y**	N

**Model 6**	N	**Y**	N	N

**Model 7**	N	**Y**	N	**Y**

**Model 8**	**Y**	**Y**	N	**Y**

Model 9**	**Y**	**Y**	Wins/Losses	**Y**

**Model 10**	**Y**	**Y**	Wins/Losses	N


**Y** indicates that a parameter was estimated for that model; **N** indicates that a parameter was not estimated for that model. * Prior variance for all parameters was set to a precise value of 2^–2^ in order to deter over-fitting. ** Winning model.

### 2.4 Statistical analyses

All analyses were performed in R or MATLAB. We first re-performed the same model assessment measures as in the original paper for the 1-year follow-up data. This included model accuracy metrics, reflecting (1) the average probability of participants’ actions across trials under the model, and (2) the average percentage of trials for which the highest probability action in the model matched the action chosen by participants (i.e., under subject-specific parameter estimates).

We next examined whether participants who did vs. did not return for the follow-up in each group differed in baseline model parameter values, symptom severity, and/or age, sex, or premorbid IQ. As in our prior study, we then ran a parametric empirical Bayes (PEB) analysis (K. J. Friston et al., 2016; Zeidman et al., 2019) using standard MATLAB routines (see software note) to assess stability of group differences over time in both the full and propensity-matched sample. PEB computes group posterior estimates in a general linear model that incorporates posterior variances of individual-level parameter estimates when assessing evidence for group-level models with and without the presence of effects of group and time (and their interaction). A further benefit of this type of hierarchical Bayesian analysis is that it is robust against concerns related to multiple comparisons (Gelman, Hill, & Yajima, 2012; Gelman & Tuerlinckx, 2000; Dienes, 2008, 2011, 2014). We specifically ran models including age, sex, the Wide Range Achievement Test (WRAT) reading score (henceforth referred to as premorbid IQ), group (SUDs versus HCs), time, and their interaction as predictor variables (see **Supplementary Materials** for further details). For consistency with frequentist analyses in our baseline study, and with analyses of model-free variables below, supplementary linear mixed effects models (LMEs) with the same predictors were also run for posterior parameter means as point estimates.

In the full sample, we then estimated the longitudinal stability of overall task performance (total wins) and individual parameter estimates between baseline and 1-year follow-up using single-measure consistency intraclass correlations that account for fixed effects across time [ICC(3, 1)]. We chose this ICC measure due to the expectation that time and/or task familiarity could plausibly influence task behavior equivalently across all participants. Although we note that these ICCs should not be interpreted as standard test-retest reliability analyses due to the lengthy time period between sessions, where true changes in participant characteristics can plausibly occur, including changes in symptom severity. To address this possibility, we also performed exploratory analyses examining the relationship between pre-post change scores in parameters and pre-post changes in DAST scores, while accounting for age, sex, and premorbid IQ.

Next, in the SUD group, we performed exploratory analyses examining whether parameter values at baseline could predict symptom severity (DAST) scores at 1-year follow-up, before and after accounting for what could be predicted from differences in baseline symptom levels, age, sex, and premorbid IQ. These analyses were performed across all SUDs, as well as when separating individuals by specific SUDs (i.e., with the exception of hallucinogen use disorders, due to insufficient sample size [N = 3]). For these analyses, and the change score analyses above, six participants in the SUDs group were removed due to floor values for DAST at baseline (i.e., due to abstinence prior to study participation), as this prevented the possibility of measurable symptom decreases. Although exploratory, we also indicate whether identified relationships survive a Bonferroni correction for multiple comparisons. We also report associated Bayes factors (BFs) for these correlations to assess the probability of the data under models with vs. without these relationships (i.e., using JZS Bayes factor analyses with default prior scales in R; BayesFactor package (Morey & Rouder, 2015; Rouder, Morey, Speckman, & Province, 2012)). To calculate these BFs, the BayesFactor package assumes noninformative priors for the population means and variances; a shifted, scaled beta (1/*rscale*,1/*rscale*) prior distribution is assumed for the linear relationship in the population (Ly, Verhagen, & Wagenmakers, 2016), with *rscale* = 1/3.

Finally, to confirm relationships seen at baseline between model parameters and model-free metrics of task behavior, we first calculated mean reaction times (RTs), trimmed using an iterative Grubbs test method to remove outliers until a distribution was found which contained no outliers at a threshold of *p* < .01; (Grubbs, 1969). This was the same method used in our prior report on the baseline data, and was done to minimize any noise in the data due to influences unrelated to the decision processes of interest, such as lapses in attention or accidental button presses. We also calculated the number of stays vs. shifts in bandit selection after win and loss outcomes. We examined the relationship between each of these model-free metrics and our model parameters to gain more insights into the meaning of observed differences. Toward this end, we examined the first and second halves of the games separately (i.e., first 7 choices vs. final 8 choices) to assess periods wherein exploration vs. exploitation would be expected to dominate. To test for consistency with our baseline findings, we also report results of LMEs assessing effects of group and time (and their interaction) on these measures when accounting for age, sex, and premorbid IQ (as well as associated Bayes factors).

As in our prior study, we note here that each of these analyses are considered exploratory, as part of the pre-defined exploratory sample of T1000 participants. Pre-registered analyses will be done to replicate all results in the confirmatory sample (i.e., the subset of the latter 500 participants of the T1000 sample meeting criteria for HC or SUD groups).

## 3. Results

### 3.1 Model comparison and accuracy

When comparing the 10 nested models (Table 4), the winning model at 1-year follow-up was the same model previously found to best explain behavior at baseline – including action precision (*a*), reward sensitivity (*c_r_*), separate learning rates for wins (*η_win_*) and losses (*η_loss_*), and insensitivity to information (*a*_0_) (protected exceedance probability = 1). On average, this model accurately predicted true actions on 63% of trials (SD = 11%); SUDs = 62% (SD = 10%), HCs = 64% (SD = 11%). The average probability assigned to participants’ true actions by this model was .57 (SD = .11); SUDs = .57 (SD = .11), HCs = .58 (SD = .11). Note that chance accuracy = 1/3. Parameter recoverability analyses showed that generative and estimated parameters for simulated behavior under this model were highly correlated for the range of parameter values observed in our participants: action precision (*r* = .80, *p* < .001), reward sensitivity (*r* = .90, *p* < .001), learning rate for wins (*r* = .91, *p* < .001), learning rate for losses (*r* = .91, *p* < .001), insensitivity to information (*r* = .79, *p* < .001). For results of further diagnostic analyses assessing model identifiability and stability of parameter estimates under different choices of prior means, see **Supplementary Materials**. In brief, results of model identifiability analyses showed that the winning model (i.e., Model 9 in Table 4) was correctly identified in model comparison when it was used to generate simulated data. However, one other model (Model 10) was also incorrectly identified as Model 9, suggesting these two models (i.e., the only models containing separate learning rates for wins and losses) may not be fully distinguishable. Results also confirmed that parameter estimates for Model 9 were highly correlated when estimated under distinct prior means, suggesting that our results are not strongly prior-dependent.

### 3.2 Longitudinal stability of group differences

When comparing individuals at baseline in each group who did vs. did not return for follow-up, those who did not return did not significantly differ in age, sex, OASIS, scores, PHQ scores, or DAST scores at baseline (in either the full or matched samples).

**Figure 2:**
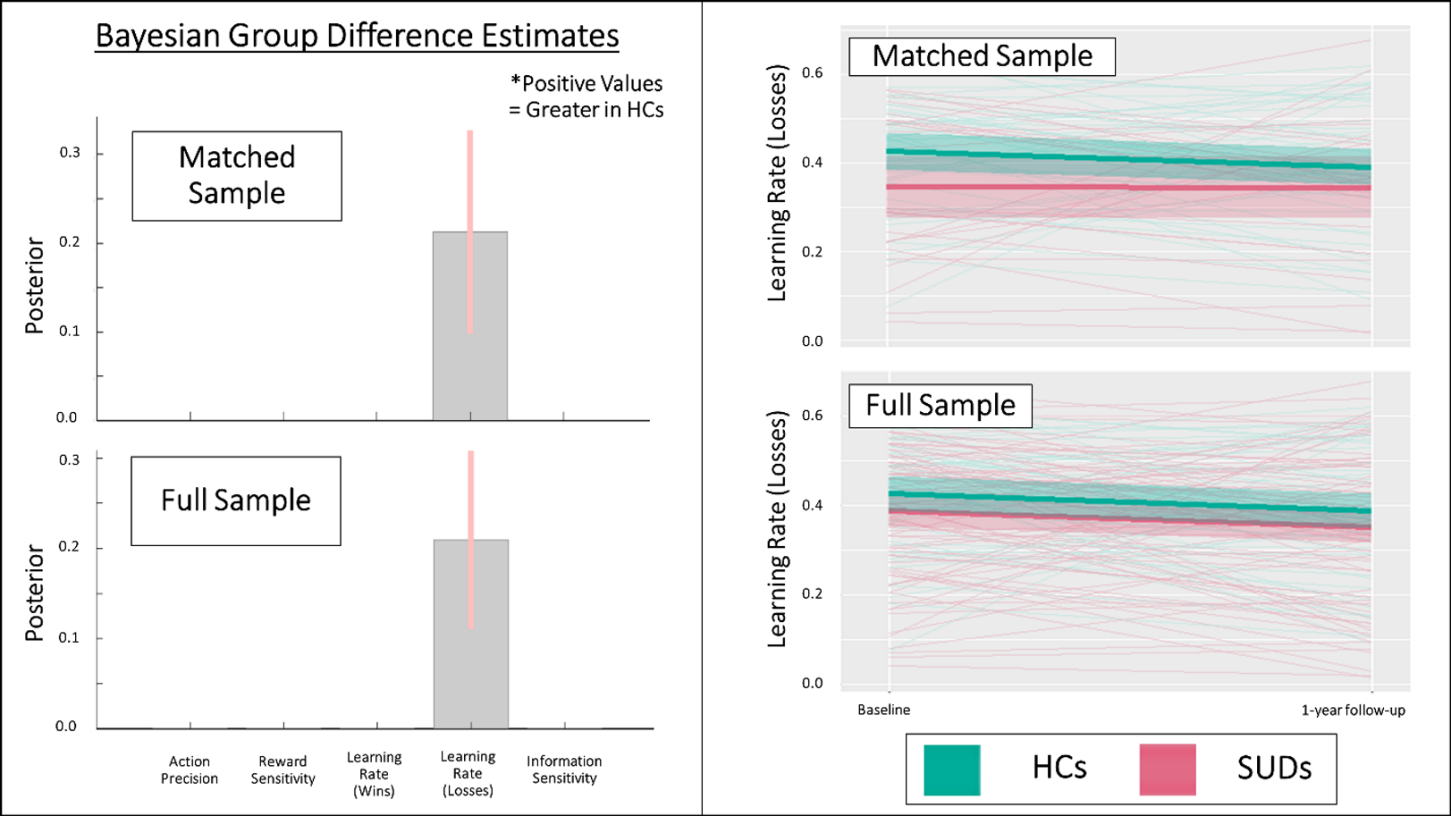
*Left*: Results of parametric empirical Bayes (PEB) analyses, showing the posterior means and variances for group difference estimates in the full and propensity-matched samples in models accounting for age, sex, and premorbid IQ. These Bayesian group comparisons confirm the differences in learning rates for losses seen at baseline. There was also a main effect of time on this learning rate, but no significant interactions between group and time, indicating the group effects were stable. No other parameters showed main effects of group. See main text for further results of these analyses. Learning rate values are in logit-space. *Right*: Spaghetti plots showing individual changes from baseline to follow-up, as well as group means and standard errors, for learning rate for losses in the full and matched samples. HCs = healthy controls, SUDs = substance use disorders.

Table 5 presents descriptive statistics for parameters by group. Bayesian (PEB) analyses testing effects on posterior distributions (means and variances) for each parameter also revealed very strong evidence for a number of effects in both the full and matched samples (posterior probability = 1 in all cases). When assessing potential effects of group, time, and their interaction (and accounting for age, sex, and baseline premorbid IQ), the model with the most evidence in both the full and matched samples included a sustained group difference in learning rate for losses from baseline to follow-up (slower in SUDs; full sample: *b* = 0.21, credible interval [CI] = [0.11, 0.31]; matched sample: *b* = 0.21, CI = [0.10, 0.33]; see Figure 2). For statistical results in analogous LMEs taking a frequentist approach, see Table 5. However, these analyses did not support a sustained difference in action precision or learning rate for wins as seen in our previous report, or a group difference in any other parameter. There were also effects of time on reward sensitivity (increases over time; full sample: *b* = 0.09, CI = [0.07, 0.11]; matched sample: *b* = 0.09, CI = [0.07, 0.12]) and learning rate for losses (decreases over time; full sample: *b* = –0.22, CI = [–0.15, –0.28]; matched sample: *b* = –0.12, CI = [–0.05, –0.20]) in both samples. There were no interactions between group and time for any parameter in the full sample. In contrast, within the matched sample, group by time interactions were present in the winning model for reward sensitivity (steeper increase over time in SUDs; *b* = –0.06, CI = [–0.03, –0.09]) and learning rates for wins (decrease over time in SUDs but increase over time in HCs; *b* = 0.17, CI = [0.09, 0.25]).

**Table 5:** Model Parameters by Group and Session (Means and Standard Deviations) as well as Results of Linear Mixed Effects Model Analyses.


FULL SAMPLE									

	TOTAL	BASELINE		FOLLOW-UP		USABLE DATA (N)	EFFECT OF CLINICAL STATUS	EFFECT OF SESSION	EFFECT OF CLINICAL STATUS/SESSION INTERACTION
	
		HCS	SUDS	HCS	SUDS				
	
	131	48	83	48	83				

**Action Precision**	2.44 (0.83)	2.57 (0.92)	2.2 (0.58)	2.71 (0.95)	2.44 (0.86)	HC: 45 SUD +: 77 Total: 122	***F*(1, 117) = 4.52** ***p* = 0.04** **η^2^ = 0.04**	***F*(1, 120) = 4.41** ***p* = 0.04** **η^2^ = 0.04**	*F*(1, 120) = 0.67 *p* = 0.41 η^2^ = 0.01

**Reward Sensitivity**	4.45 (1.49)	4.3 (1.47)	4.25 (1.5)	4.5 (1.45)	4.71 (1.49)	HC: 45 SUD +: 77 Total: 122	*F*(1, 117) = 0.98 *p* = 0.32 η^2^ = 0.01	***F*(1, 120) = 12.74** ***p* < 0.001** **η^2^ = 0.1**	*F*(1, 120) = 1.04 *p* = 0.31 η^2^ = 0.01

**Learning Rate (Wins)**	0.5 (0.13)	0.47 (0.12)	0.5 (0.13)	0.49 (0.13)	0.51 (0.15)	HC: 45 SUD +: 77 Total: 122	***F*(1, 117) = 5.41** ***p* = 0.02** **η^2^ = 0.04**	***F*(1, 120) = 3.86** ***p* = 0.05** **η^2^ = 0.03**	*F*(1, 120) = 0.01 *p* = 0.93 η^2^ = 0

**Learning Rate (Losses)**	0.38 (0.15)	0.43 (0.13)	0.39 (0.15)	0.39 (0.15)	0.35 (0.16)	HC: 45 SUD +: 77 Total: 122	***F*(1, 117) = 6.45** ***p* = 0.01** **η^2^ = 0.05**	***F*(1, 120) = 8.68** ***p* = 0.004** **η^2^ = 0.07**	*F*(1, 120) = 0.14 *p* = 0.7 η^2^ = 0

**Information Insensitivity**	0.78 (0.28)	0.72 (0.27)	0.79 (0.29)	0.76 (0.31)	0.82 (0.25)	HC: 45 SUD +: 77 Total: 122	*F*(1, 117) = 3.63 *p* = 0.06 η^2^ = 0.03	*F*(1, 120) = 1.54 *p* = 0.22 η^2^ = 0.01	*F*(1, 120) = 0.27 *p* = 0.6 η^2^ = 0

**PROPENSITY MATCHED**									

	**TOTAL**	**BASELINE**		**FOLLOW-UP**		**USABLE DATA (N)**	**EFFECT OF CLINICAL STATUS**	**EFFECT OF SESSION**	**EFFECT OF CLINICAL STATUS/SESSION INTERACTION**
	
		**HCS**	**SUDS**	**HCS**	**SUDS**				
	
	**70**	**45**	**25**	**45**	**25**				

**Action Precision**	2.51 (0.89)	2.59 (0.94)	2.12 (0.58)	2.67 (0.94)	2.51 (0.9)	HC: 45 SUD +: 25 Total: 70	*F*(1, 65) = 2.43 *p* = 0.12 η^2^ = 0.04	*F*(1, 68) = 2.86 *p* = 0.1 η^2^ = 0.04	*F*(1, 68) = 1.63 *p* = 0.21 η^2^ = 0.02

**Reward Sensitivity**	4.4 (1.51)	4.23 (1.47)	4.17 (1.63)	4.51 (1.49)	4.72 (1.52)	HC: 45 SUD +: 25 Total: 70	*F*(1, 65) = 0.02 *p* = 0.88 η^2^ = 0	***F*(1, 68) = 4.84** ***p* = 0.03** **η^2^ = 0.07**	*F*(1, 68) = 0.59 *p* = 0.45 η^2^ = 0.01

**Learning Rate (Wins)**	0.49 (0.13)	0.46 (0.12)	0.53 (0.1)	0.49 (0.13)	0.51 (0.16)	HC: 45 SUD +: 25 Total: 70	*F*(1, 65) = 2.56 *p* = 0.11 η^2^ = 0.04	*F*(1, 68) = 0.31 *p* = 0.58 η^2^ = 0	*F*(1, 68) = 1.48 *p* = 0.23 η^2^ = 0.02

**Learning Rate (Losses)**	0.39 (0.15)	0.43 (0.13)	0.35 (0.17)	0.39 (0.14)	0.34 (0.18)	HC: 45 SUD +: 25 Total: 70	***F*(1, 65) = 4.32** ***p* = 0.04** **η^2^ = 0.06**	*F*(1, 68) = 1.33 *p* = 0.25 η^2^ = 0.02	*F*(1, 68) = 0.65 *p* = 0.42 η^2^ = 0.01

**Information Insensitivity**	0.77 (0.29)	0.73 (0.27)	0.82 (0.33)	0.75 (0.28)	0.86 (0.28)	HC: 45 SUD +: 25 Total: 70	*F*(1, 65) = 3.23 *p* = 0.08 η^2^ = 0.05	*F*(1, 68) = 0.35 *p* = 0.56 η^2^ = 0.01	*F*(1, 68) = 0.07 *p* = 0.8 η^2^ = 0


* Analyses are reported using results from LMEs accounting for age, sex, and premorbid IQ (WRAT). Significant effects are bolded.

There were also effects of age, sex, and premorbid IQ on some parameters. In the full sample: (1) age was negatively associated with action precision (*b* = –0.02, CI = [–0.02, –0.03]) and positively associated with reward sensitivity (*b* = 0.03, CI = [0.02, 0.03]), (2) learning rate for wins was faster in males (*b* = 0.17, CI = [0.08, 0.26]), and (3) higher premorbid IQ was associated with slower learning rate for losses (*b* = –0.05, CI = [–0.04, –0.07]). In the matched sample, reward sensitivity was greater in males (*b* = 0.19, CI = [0.15, 0.24]) and premorbid IQ was positively associated with action precision (*b* = 0.02, CI = [0.01, 0.03]).

Additional PEB analyses focused only on 1-year follow-up data (i.e., analogous to those reported in our baseline study, accounting for age, sex, and premorbid IQ) also showed positive evidence for the group difference in learning rate for losses in both the full sample (posterior probability = .83; *b* = 0.22, CI = [0.001, 0.43]) and matched sample (posterior probability = .93; *b* = 0.28, CI = [0.07, 0.48]). For plots of each parameter by group and time in both samples, see **Supplementary Figure S2**. For plots of the additional PEB results (illustrating effect sizes) not shown in Figure 2, see **Supplementary Figure S3**. For consistency with frequentist analyses in our baseline study, Table 5 also presents effects of group, session, and their interaction within LMEs predicting the posterior parameter means (with the same additional predictors as the PEB models). Findings were largely consistent with the Bayesian results. However, significant group effects were also present in action precision and learning rate for wins in the full sample (mirroring our previously reported baseline results). Linear models equivalent to those in our baseline paper also supported PEB results in showing significantly slower learning rates for losses in SUDs than HCs when only comparing groups at follow-up (full sample: *t*(117) = 2.137, *p* = .03, *d* = 0.40), but showed no other significant differences for other parameters.

### 3.3 Individual-level parameter stability

**Figure 3:**
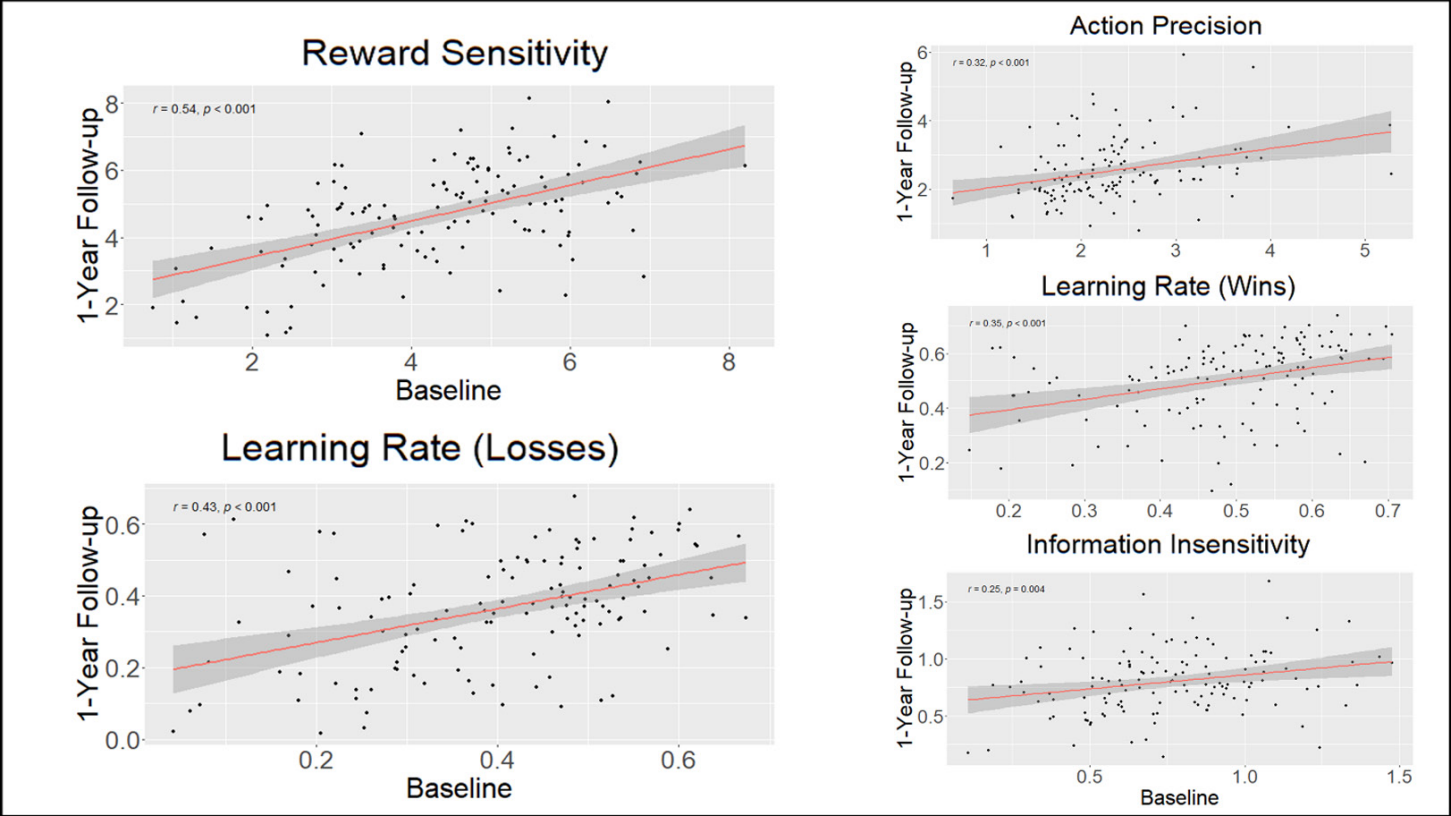
Correlations between computational parameters at baseline and 1-year follow-up.

The ICCs for task performance and parameters between baseline and 1-year follow-up were poor to moderate (see Table 6 and Figure 3), with the highest values across all participants for reward sensitivity (ICC = .54) and learning rate for losses (ICC = .43). With the exception of action precision and total wins, SUDs tended to have numerically higher ICCs than HCs. Task performance (total wins) showed the lowest stability over time across participants (ICC = .15), driven by a non-significant association between baseline and follow-up in the SUD group.

**Table 6:** Intra-class correlations between baseline and 1-year follow-up (full sample).


	GROUP	ICC(3, 1)	*p*

**Total wins**	All	.15	.05

	HCs	.27	.03

	SUDs	.08	.23

***a* (action precision)**	All	.32	<.001

	HCs	.45	<.001

	SUDs	.15	.09

***c*** ***r* (reward sensitivity)**	All	.54	<.001

	HCs	.48	<.001

	SUDs	.58	<.001

*η_**win**_* **(learning rate for wins)**	All	.35	<.001

	HCs	.28	.03

	SUDs	.37	<.001

*η_**loss**_* **(learning rate for losses)**	All	.43	<.001

	HCs	.35	.007

	SUDs	.45	<.001

***a*_0_** **(insensitivity to information)**	All	.25	.002

	HCs	.24	.05

	SUDs	.25	.01


**Figure 4:**
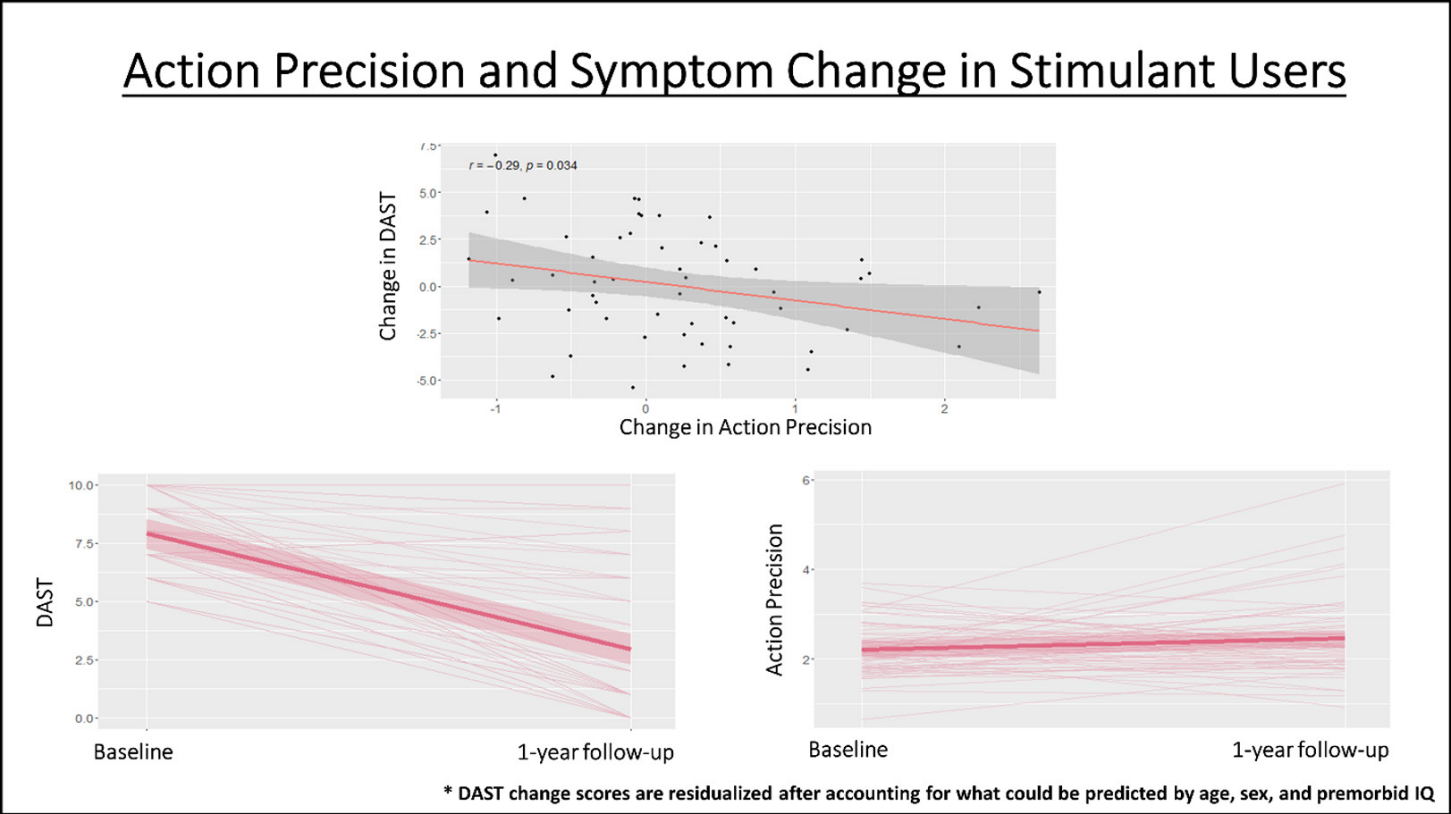
*Top*: Negative correlation in stimulant users (full sample) between pre-to-post changes in action precision and pre-to-post changes in symptom severity (DAST). *Bottom*: Illustration of individual pre-to-post changes in DAST scores and action precision (as well as group mean and SE). As can be seen, DAST scores tend to decrease and action precision tends to increase, but with notable individual differences in each. DAST change scores account for what could already be predicted based on age, sex, and premorbid IQ. However, we note that this correlation did not survive correction for multiple comparisons and will need to be replicated in future work.

There were no significant associations between pre-post changes in DAST scores and pre-post changes in parameters across all SUDs. When examining specific SUDs separately, both stimulant and opioid users showed an association between pre-post changes in DAST scores and pre-post changes in action precision. In stimulant users, this correlation was *r* = –.28 (*p* = .03, BF = 2.44), and this remained unchanged after accounting for the relationship between DAST changes and age, sex, and premorbid IQ scores (*r* = –.29, *p* = .03, BF = 2.35; see Figure 4). In opioid users, this correlation was *r* = –.34 (*p* = .07, BF = 1.65), and this became significant after accounting for the relationship between DAST changes and age, sex, and premorbid IQ scores (*r* = –.38, *p* = .046, BF = 2.18). No other associations were found (see **Supplementary Figure S4** for specific values). None of these relationships remained significant when correcting for multiple comparisons.

### 3.4 Symptom Change Prediction

**Figure 5:**
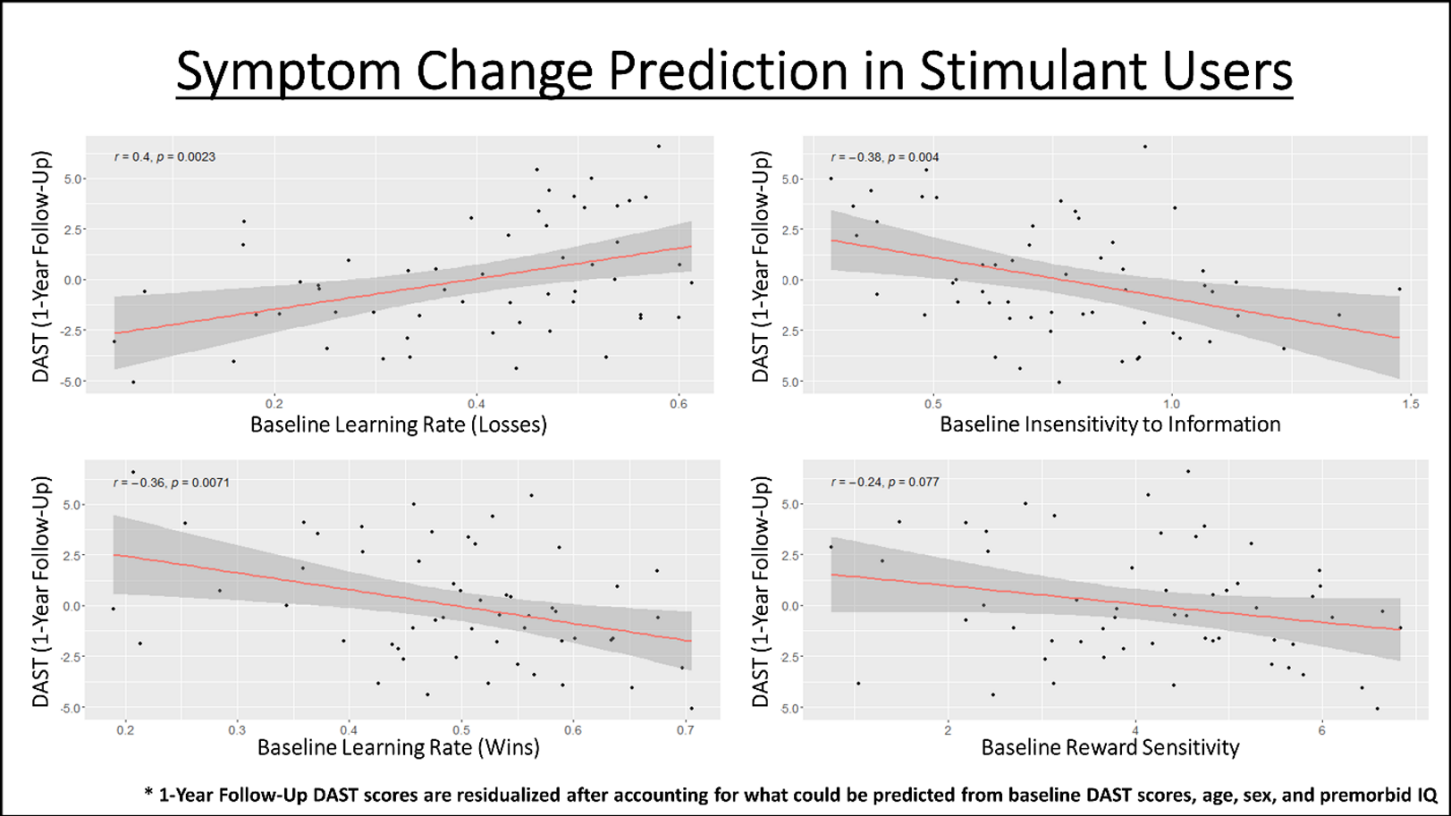
Predictive relationships in stimulant users (full sample) between baseline model parameters and symptom severity at 1-year follow-up, after accounting for what could already be predicted based on age, sex, and premorbid IQ. The *p*-values shown here are uncorrected, but the relationships with learning rates and insensitivity to information survive correction for 6 comparisons (i.e., one per SUD group tested; corrected threshold of *p* < .0083). No relationship survives a more conservative correction for 30 comparisons (i.e., accounting for 5 parameters tested in each SUD group; corrected threshold of *p* < .0017).

In the full sample of substance users, no significant predictive relationships were found between baseline model parameters and DAST scores at 1-year follow-up (after accounting for baseline DAST scores, with or without accounting for age, sex, and premorbid IQ). However, when restricting the sample to stimulant users in subsequent exploratory analyses, we observed a significantly positive predictive relationship between baseline learning rates for losses and DAST scores at 1-year follow-up (*r* = .33, *p* = .01, BF = 5.91), which became stronger after accounting for what could be predicted by age, sex, and premorbid IQ (*r* = .4, *p* = .002, BF = 20.99; see Figure 5). Significant negative predictive relationships were also found with baseline learning rates for wins (*r* = –.29, *p* = .03, BF = 2.89) and insensitivity to information (*r* = –.36, *p* = .005, BF = 11.46), which each also became stronger after accounting for age, sex, and premorbid IQ (respectively: *r* = –.36, *p* = .007, BF = 8.18; *r* = –.38, *p* = .004, BF = 13.08; see Figure 5). Each of the results accounting for age, sex, and IQ survived Bonferroni correction for 6 comparisons (i.e., for assessing relationships in the full sample and the 5 specific SUD subsamples; corrected threshold: *p* < .0083). However, none remain significant if using a more conservative correction for 30 comparisons (i.e., 5 parameters within the total sample and in each subsample; corrected threshold: *p* < .0017). When restricting analyses to opioid users, we observed a significantly negative predictive relationship between baseline information insensitivity and DAST scores at 1-year follow-up (*r* = –.43, *p* = .02, BF = 4.10), which weakened after accounting for what could be predicted by age, sex, and premorbid IQ (*r* = –.35, *p* = .07, BF = 1.66). However, this relationship did not survive correction for multiple comparisons. When restricting analyses to alcohol users, there was a trending negative relationship with information insensitivity (r = –.37, p = .07), which weakened after accounting for what could be predicted by age, sex, and premorbid IQ (r = –.26, p = .22). No other notable relationships were observed (see **Supplementary Figure S4** for specific values).

Although not a part of our initial hypotheses, for the interested reader (and for the purpose of future hypothesis generation) we report subsequent post-hoc exploratory analyses within **Supplementary Materials** examining possible relationships between model parameters and symptom severity at follow-up. These analyses did not reveal significant results (although suggestive trends were present in some cases).

### 3.5 Comparison to model-free measures

Table 7 lists descriptive statistics by group and time in model-free behavioral measures (total wins, win/lose stay/shift choices, and RTs). This table also shows results of LMEs assessing the main effects and interactions between group and time, while accounting for age, sex, and premorbid IQ. In **Supplementary Tables S1–2**, results are further divided into sets derived from early trials (i.e., where information-seeking should be high; choices 2–7 per game), and late trials (i.e., where reward-seeking would be expected to dominate; subsequent 8 choices). Most notably, these results together indicated that, relative to HCs, SUDs showed a larger number of lose-stay choices across time (driven by choices in early trials) in both the propensity-matched and full samples. They also showed a smaller number of lose-shift choices across time (present in both early and late trials) in the full sample. A follow-up LME in the full sample testing for main effects and interactions between clinical group and early vs. late trial phase in predicting number of lose-stay choices confirmed the presence of a significant interaction between group and trial phase (*F*(1, 462) = 5.15, *p* = 0.02), as well as a main effect of trial phase (*F*(1, 462) = 59.88, *p* < 0.001; a greater number of lose-stay choices in late trials). Although it showed a similar numerical trend (see **Table S2**), this interaction was not significant in the matched sample.

**Table 7:** Model-Free Task Measures by Group and Session (Means and Standard Deviations).


FULL SAMPLE									

	TOTAL	BASELINE		FOLLOW-UP		USABLE DATA (N)	EFFECT OF CLINICAL STATUS	EFFECT OF SESSION	EFFECT OF CLINICAL STATUS/SESSION INTERACTION
	
		HCS	SUDS	HCS	SUDS				
	
	131	48	83	48	83				

Wins	181.1 (12.71)	182.83 (12.19)	178.75 (12.79)	182.27 (12.14)	181.78 (13.12)	HC: 45 SUD +: 77 Total: 122	*F*(1, 117) = 1.97 *p* = 0.16 η^2^ = 0.02	*F*(1, 120) = 1.33 *p* = 0.25 η^2^ = 0.01	*F*(1, 120) = 0.74 *p* = 0.39 η^2^ = 0.01

Mean Reaction Time	0.56 (0.25)	0.62 (0.24)	0.61 (0.27)	0.53 (0.23)	0.5 (0.22)	HC: 45 SUD +: 77 Total: 122	*F*(1, 117) = 3.46 *p* = 0.07 η^2^ = 0.03	***F*(1, 120) = 29.56** ***p* < 0.001** **η^2^ = 0.2**	*F*(1, 120) = 0.6 *p* = 0.44 η^2^ = 0.01

Win/Stay	134.28 (33.91)	133.5 (32.91)	131.63 (36.63)	131.21 (31.49)	139.17 (33.03)	HC: 45 SUD +: 77 Total: 122	*F*(1, 117) = 0.27 *p* = 0.6 η^2^ = 0	*F*(1, 120) = 2.61 *p* = 0.11 η^2^ = 0.02	*F*(1, 120) = 1.99 *p* = 0.16 η^2^ = 0.02

Win/Shift	35.08 (28.17)	37.88 (28.11)	35.46 (29.6)	39.06 (25.85)	30.8 (27.94)	HC: 45 SUD +: 77 Total: 122	*F*(1, 117) = 1.42 *p* = 0.24 η^2^ = 0.01	*F*(1, 120) = 2.15 *p* = 0.15 η^2^ = 0.02	*F*(1, 120) = 1.24 *p* = 0.27 η^2^ = 0.01

Lose/Stay	47.11 (29.45)	39.33 (25.51)	46.42 (30.11)	45.98 (24.45)	52.96 (32.66)	HC: 45 SUD +: 77 Total: 122	***F*(1, 117) = 7.21** ***p* = 0.008** **η^2^ = 0.06**	***F*(1, 120) = 9.16** ***p* = 0.003** **η^2^ = 0.07**	*F*(1, 120) = 0.06 *p* = 0.8 η^2^ = 0

Lose/Shift	83.52 (30.99)	89.29 (27.39)	86.49 (32.07)	83.75 (27.07)	77.07 (33.27)	HC: 45 SUD +: 77 Total: 122	***F*(1, 117) = 4.08** ***p* = 0.05** **η^2^ = 0.03**	***F*(1, 120) = 12.7** ***p* < 0.001** **η^2^ = 0.1**	*F*(1, 120) = 0.79 *p* = 0.38 η^2^ = 0.01

**PROPENSITY-MATCHED**									

	**TOTAL**	**BASELINE**		**FOLLOW-UP**		**USABLE DATA (N)**	**EFFECT OF CLINICAL STATUS**	**EFFECT OF SESSION**	**EFFECT OF CLINICAL STATUS/SESSION INTERACTION**
	
		**HCS**	**SUDS**	**HCS**	**SUDS**				
	
	**70**	**45**	**25**	**45**	**25**				

Wins	180.76 (12.56)	182.4 (12.46)	177.92 (11.15)	182.44 (11.95)	177.64 (14.65)	HC: 45 SUD +: 25 Total: 70	*F*(1, 65) = 3.44 *p* = 0.07 η^2^ = 0.05	*F*(1, 68) = 0 *p* = 0.97 η^2^ = 0	*F*(1, 68) = 0.01 *p* = 0.93 η^2^ = 0

Mean Reaction Time	0.55 (0.23)	0.62 (0.25)	0.57 (0.21)	0.53 (0.23)	0.47 (0.17)	HC: 45 SUD +: 25 Total: 70	*F*(1, 65) = 1.87 *p* = 0.18 η^2^ = 0.03	***F*(1, 68) = 14.39** ***p* < 0.001** **η^2^ = 0.17**	*F*(1, 68) = 0.04 *p* = 0.85 η^2^ = 0

Win/Stay	132.44 (32.63)	132.96 (33.22)	129.64 (38.41)	132.24 (32.13)	134.68 (27.58)	HC: 45 SUD +: 25 Total: 70	*F*(1, 65) = 0 *p* = 0.97 η^2^ = 0	*F*(1, 68) = 0.09 *p* = 0.76 η^2^ = 0	*F*(1, 68) = 0.39 *p* = 0.54 η^2^ = 0.01

Win/Shift	36.78 (27.93)	38.11 (28.05)	37.04 (33.09)	38.09 (26.38)	31.76 (25.9)	HC: 45 SUD +: 25 Total: 70	*F*(1, 65) = 0.46 *p* = 0.5 η^2^ = 0.01	*F*(1, 68) = 0.28 *p* = 0.6 η^2^ = 0	*F*(1, 68) = 0.49 *p* = 0.49 η^2^ = 0.01

Lose/Stay	46.19 (29.26)	38.31 (25.88)	51 (30.01)	45.22 (24.7)	57.32 (37.94)	HC: 45 SUD +: 25 Total: 70	***F*(1, 65) = 4.56** ***p* = 0.04** **η^2^ = 0.07**	***F*(1, 68) = 3.97** ***p* = 0.05** **η^2^ = 0.06**	*F*(1, 68) = 0.01 *p* = 0.93 η^2^ = 0

Lose/Shift	84.59 (29.69)	90.62 (27.71)	82.32 (32.22)	84.44 (27.78)	76.24 (33.13)	HC: 45 SUD +: 25 Total: 70	*F*(1, 65) = 2.04 *p* = 0.16 η^2^ = 0.03	*F*(1, 68) = 3.13 *p* = 0.08 η^2^ = 0.04	*F*(1, 68) = 0 *p* = 0.99 η^2^ = 0


* Analyses are reported using results from LMEs accounting for age, sex, and premorbid IQ (WRAT). Significant effects are bolded.

**Supplementary Figure S5** shows the correlations between model parameters and model-free measures at 1-year follow-up. As can be seen there, results strongly resembled those previously found in our baseline study. First, there was a complex pattern of relationships with win/lose stay/shift behavior in which reward sensitivity and information insensitivity promoted stay behaviors generally, action precision promoted win-stay choices on late trials, and learning rates had relationships with all types of choices in expected directions, but with the strongest relationship to stays vs. switches on loss trials. Number of wins only showed associations with reward sensitivity and action precision (positive relationship). This relationship was notably (numerically) stronger on late trials in each game. RTs were faster in those with higher reward sensitivity, information sensitivity, and learning rate for wins, and slower in those with higher learning rate for losses (*p*s < .001 and BFs > 100 in all cases).

## 4. Discussion

In this study, we evaluated the longitudinal stability of both individual- and group-level differences between HCs and SUDs in computational measures of learning and decision-making over a 1-year period. We also examined whether these computational measures could predict changes in symptom severity over time. At the group level, both Bayesian and frequentist analyses showed that a slower learning rate for losses in SUDs (previously observed at baseline (Smith, Schwartenbeck, et al., 2020)) was stable over the 1-year period. Comparison to descriptive measures suggested that this (in part) tracked the fact that SUDs tended to continue with the same decision strategy after incurring a loss (primarily on early trials). This appears consistent with previous results showing associations between SUDs and difficulty avoiding punishment (Myers et al., 2017), diminished responses to negative stimuli (Hester, Bell, Foxe, & Garavan, 2013; Simons & Arens, 2007; Simons, Dvorak, & Batien, 2008; Stewart et al., 2014), reduced sensitivity to losses (Ahn et al., 2014), and a lower impact of large losses on future choices (Petry, Bickel, & Arnett, 1998). Importantly, it could help explain why substance use continues despite negative life consequences. As changes in this learning rate did not correspond to symptom changes over time, it might more plausibly act as a pre-existing (trait) vulnerability factor. For example, those with a greater tendency to persist in a pattern of behavior despite negative outcomes could be more likely to engage in substance use a sufficient number of times to promote addiction. On the other hand, substance misuse over time could lead to less sensitivity to negative outcomes regardless of future symptom change.

At the individual level, we found that some parameters showed moderate stability while others showed poor stability. The two most (moderately) stable parameters were learning rate for losses and reward sensitivity. As the former reflected the primary group differentiator, this further supports its potential role as a pre-existing vulnerability factor, which could act as an adjunct assessment of risk independent of self-report. While parameter estimation error could partly account for these attenuated relationships, we also examined whether the lower levels of stability we observed might be due to associations with individual differences in symptom changes. While not present across all SUDs, in stimulant and opioid users we found that larger reductions in symptom severity were associated with larger increases in action precision, which could suggest this parameter reflects evolving aspects of the disease process (although this will require replication before being afforded high confidence, as it did not survive correction for multiple comparisons). In our baseline study, SUDs showed significantly lower action precision than HCs, while this difference was no longer present at follow-up. This was due to increased action precision over time in SUDs – mirroring the overall reduction in symptom severity at follow-up. Given this pattern, future research should assess whether action precision might act as an objective measure of treatment progress.

When evaluating the predictive utility of baseline parameters, we did not find significant results across all SUDs. However, we did observe significant predictive relationships when restricting analyses to specific SUDs. Namely, we found that symptom severity at follow-up in stimulant users was predicted by baseline learning rate for losses (positive relationship), and also by information insensitivity and learning rate for wins (negative relationships); although we note that these relationships did not survive the most conservative approach to correction for multiple comparisons. Opioid users’ symptoms at follow-up showed a similar negative relationship with baseline information insensitivity, but this did not survive correction for multiple comparisons. If replicated in an independent confirmatory sample, assessment of these measures at treatment onset might therefore offer additional information about which patients will be more resistant to improvement over time. This represents another important topic for future research.

Despite SUDs showing slower learning from losses (and some evidence for faster learning from wins in frequentist analyses) at the group level, stimulant users with the slowest learning rates from losses (and fastest learning rates from wins) had better outcomes at follow-up. Also, despite (numerically) greater insensitivity to information in SUDs at the group level, both stimulant and opioid users with the greatest insensitivity also had lower symptoms at follow-up. One might speculate that, upon initiating abstinence, a slower learning rate from negative consequences could attenuate avoidance (akin to reducing lose/switch decisions) of the uncomfortable aspects of the recovery process (e.g., withdrawal, reflection on poor life circumstances in therapy, etc.) and allow a person to persist through a difficult situation without resorting to maladaptive coping mechanisms. However, such possibilities would require further investigation. Greater information insensitivity is also theoretically associated with reduced subjective uncertainty and greater confidence in expected action outcomes. In the right (e.g., therapeutic) circumstances, this could perhaps also play a role in facilitating recovery. However, there are also plausible ways in which these differences might be expected to have opposing effects as well. Independent of their predictive value, future research should therefore further address the theoretical significance and correct interpretation of these relationships, as they could speak to important components of decision-making mechanisms in SUDs that deserve attention as possible targets of behavioral interventions (Verdejo-Garcia et al., 2018; Verdejo-Garcia, Garcia-Fernandez, & Dom, 2019).

Although representative of the population (and therefore potentially more informative in real-world clinical settings), one limitation of this study is the heterogeneity of our SUD group. Several secondary analyses in our baseline study addressed some related concerns, but they nonetheless constrain interpretability here. For example, the predictive relationships we found separately in stimulant and opioid users suggest that other SUDs (e.g., cannabis, sedatives) may have had confounding effects; but samples of individuals with each of these disorders in isolation would be needed to definitively answer this question. Another issue is that, although we did not identify differences in those who did versus did not return for the follow-up visit, drop-out nonetheless reduced the statistical power available for our analyses and could still limit the generalizability of our results. We plan to address these issues further in the confirmatory dataset presently set aside within the T1000 project to replicate these results. A final issue worth highlighting is that model identifiability analyses suggested that model comparison was limited in its ability to distinguish the winning model from a model that did not include the insensitivity to information parameter. With this limitation in mind, the presence of distinct learning rates for wins and losses did appear identifiable, which supports the validity of our primary results.

In summary, we found that individuals with SUDs showed stable reductions in learning from losses relative to HCs over a 1-year period. Individual-level parameter stability was poor-to-moderate, and in some cases appeared to be attenuated by symptom changes. Finally, multiple model parameters at baseline showed potential predictive utility with respect to symptom changes over time. These results hold promise in the development of adjunct computational assessment tools for predicting symptom evolution and perhaps treatment progress, which could inform treatment decisions.

## Software Note

All model simulations, model comparison, and parametric empirical Bayes analyses were implemented using standard routines (**spm_MDP_VB_X.m, spm_BMS.m, spm_dcm_peb.m, spm_dcm_peb_bmc.m**) that are available as MATLAB code in the latest version of SPM academic software: http://www.fil.ion.ucl.ac.uk/spm/. For the specific code used to build the three-armed bandit task model and fit parameters to data, see: https://github.com/rssmith33/3-armed_bandit_task_model.

## Additional Files

The additional files for this article can be found as follows:

10.5334/cpsy.85.s1 Supplementary Materials. Supplementary modeling methods, analyses, and figures.

10.5334/cpsy.85.s2 Supplementary Code. Study Data.

10.5334/cpsy.85.s3 Supplementary MATLAB code. Study Data.
