# Supplementary material for: Slower Learning Rates from Negative Outcomes in Substance Use Disorder over a 1-Year Period and Their Potential Predictive Utility
Source: Comput Psychiatr. 2022 Jun 8;6(1):117–41. doi: 10.5334/cpsy.85 (PMC11104312; doi:10.5334/cpsy.85)
Supplement: Supplementary Materials. — Supplementary modeling methods, analyses, and figures. [file cpsy-6-1-85-s1.pdf]

Supplementary Materials

Computational modeling details

To model behavior on the task, we adopted a Markov decision process (MDP) model under the active inference framework (see main text **Figure 1**); for more details about the structure and mathematics of this class of models, see (Friston et al., 2017; Friston et al., 2017; Parr and Friston, 2017; Smith et al., 2022). This approach requires creating a model with specific sets of possible observations (o_t^m), hidden states (s_t) that cause those observations, and available actions (policies; π). In our model, there were two types of observations (modalities; m) that could be made at each time point (t). In the first modality (o_t^{reward}), the participant could make a "starting" observation, and then observe either a win or a loss. In the second modality (o_t^{choice}), the participant could observe the action that was chosen. Hidden states in the model included a "starting" state as well as the state of having chosen each of the three options. Policies included the three available choices on each trial.

The dependencies between these variables are described by sets of matrices. One set of matrices **A** encodes the way hidden states generate observations, $p(o_t^m|s_t)$. In our model, **A** defines the probability of observing a win vs. a loss given the state of having chosen each option:

$$\mathbf{A}(reward) = p\left(o_t^{reward}\middle|s_t^{choice}\right) = \begin{bmatrix} 1 & 0 & 0 & 0 \\ 0 & r_1 & r_2 & r_3 \\ 0 & 1-r_1 & 1-r_2 & 1-r_3 \end{bmatrix}$$

Here, columns indicate (from left to right) the starting state and choices 1, 2, and 3; the rows (from top to bottom) indicate the starting observation, observing a win, or observing a loss. The values of r_1, r_2, r_3 are the true reward probabilities for each choice. There was also a second **A**-matrix mapping each choice state to the observation of that choice, which was set as an identity matrix (i.e., there was no uncertainty in the choice a participant made).

A set of matrices $\mathbf{B}_\pi$ encode state transition probabilities under each policy, $p(s_{t+1}|s_t, \pi)$. In our model, these defined the transition from the "starting state" to the state of having chosen each possible option under each respective policy. Here, the transition probabilities were simply a deterministic mapping based on participants' choices, such that, for example, $p(s_{choice\ 1}|s_{start}, \pi_{choice\ 1}) = 1$ and 0 for all other transitions.

A set of vectors C encode the subjective reward value of each observation in each modality at each time point. In our model, a value of 0 was fixed for all observations except for observing a win. The value for observing a win was estimated based on participant behavior as an index of reward sensitivity (c_r):

$$C\ (reward) = [0\ c_r\ 0]^{\mathrm{T}}$$

$$\ln p(o) = \ln\big(\sigma(C)\big)$$

The subjective reward values for different observations are formally specified in terms of a participant's *log-expectations*. The symbol σ indicates a softmax (normalized exponential) function that first transforms the values in C into a proper probability distribution, such that higher values for c_r are formally assigned higher prior probabilities (corresponding to greater subjective rewardingness of a win). This distribution is then converted into log probabilities. Higher values of c_r reduce information-seeking (by effectively increasing the weight of the reward-seeking term in the expected free energy; shown below).

A vector $D = [1\ 0\ 0\ 0]^{\mathrm{T}}$specified a prior over initial states, such that the participant always started in an undecided starting state at the beginning of each trial.

Action policies (π) are assigned values based on a quantity called expected free energy (G). When there is no uncertainty about choice states (i.e., no uncertainty about one's choice on a trial), as is true in our task, the expected free energy can be written as:

$$G_\pi = -E_{q(o,s,\mathbf{A}|\pi)}[\ln q(\mathbf{A}|s,o,\pi) - \ln q(\mathbf{A})] - E_{q(o,s,\mathbf{A}|\pi)}[\ln p(o)]$$

This quantity assigns higher values to actions that are expected to simultaneously maximize information gain and reward. The first term on the right corresponds to information gain. Note that the variable $q()$ is used to denote the participant's approximate posterior beliefs. Large values for this first term indicate the expectation that beliefs about reward probabilities will undergo a large change (i.e., that a lot will be learned about these probabilities) given a choice of policy. The second term on the right motivates reward-seeking, by maximizing $\ln p(o)$. Because these terms are subtracted, policies associated with high expected reward and high expected information gain will be assigned a lower expected free energy. This can also be seen more explicitly when expected free energy is shown in the following equivalent form that is cast in terms of model variables (for a full derivation, see (Da Costa et al., 2020)):

$$G_\pi = \sum_t \left(o_{\pi,t} \cdot \left(ln\, o_{\pi,t} - ln\, C\right) - \mathbf{A}s_{\pi,t} \cdot \mathbf{W}s_{\pi,t}\right)$$

$$\mathbf{W} := \frac{1}{2}(\mathbf{a}^{\odot(-1)} - \mathbf{a}_{sums}^{\odot(-1)})$$

In the first equation, it can be seen that policies will have higher value if 1) they minimize the divergence between predicted and preferred outcomes – $o_{\pi,t} \cdot \left(ln\, o_{\pi,t} - ln\, C\right)$ – which can be thought of as maximizing reward probability; and if 2) they seek out states expected to provide the most informative observations about the reward probabilities – $\mathbf{A}s_{\pi,t} \cdot \mathbf{W}s_{\pi,t}$ – which can be thought of as goal-directed information-seeking. In the second equation, the variable **a** within **W** denotes the current concentration parameters of Dirichlet priors over reward probabilities associated with the **A** matrix. The $:=$ symbol indicates that two things are defined to be equivalent, and the $\odot$ symbol indicates the element-wise power (i.e., separately raising each element in a matrix to the power of some number). The term $\mathbf{a}_{sums}$ is a matrix of the same size as **a** where each entry within a column corresponds to the sum of the values of the associated column in **a**. At the start of each game, **a** is as follows**:**

:

$$P(\mathbf{A}) = Dirichlet(\mathbf{a})$$

$$\mathbf{a}\ (reward) = P(o_{reward}|s_{choice}) = \begin{bmatrix} 1 & 0 & 0 & 0 \\ 0 & a_0 & a_0 & a_0 \\ 0 & a_0 & a_0 & a_0 \end{bmatrix}$$

The value of a_0 – the *insensitivity to information* parameter – is the starting value for beliefs about these reward probabilities. These beliefs always start by making up an uninformative (flat) distribution, but higher starting values (e.g., 5 vs. 0.5) effectively down-weight the information-gain term in the expected free energy – leading to an insensitivity to the need for information. Put another way, when information insensitivity (a_0) is high, the need for information-seeking is low a priori. This parameter was estimated for each participant.

The values within $\mathbf{a}\ (reward)$ are then updated as follows:

$$\mathbf{a}_{trial} = \mathbf{a}_{trial-1} + \eta \times \sum_{\tau} o_t \otimes \boldsymbol{s}_t$$

Here $\otimes$ indicates the cross-product and $\boldsymbol{s}$ is the posterior belief over choice states (i.e., the belief about which option was chosen). The variable η is the *learning rate*, which controls the magnitude of updates in $\mathbf{a}$ after each observation. This rate can also differ for different observations. Here there were separate learning rates for observing wins vs. losses. A higher learning rate will tend to promote a faster switch to reward-seeking behavior.

Once expected free energy is evaluated, the probability of selecting a policy is:

$$p(\pi) = \sigma(-G_{\pi})$$

Here, a softmax function (σ) transforms the negative expected free energies into a proper probability distribution, such that policies with lower expected free energies are assigned higher probabilities.

A final parameter pertains to choice stochasticity. Active inference naturally distinguishes between uncertainty reduction due to goal-directed, strategic information-seeking (driven by the information gain term in expected free energy) and that due to stochastic choice. The latter approach to gaining information through stochastic choice can be accounted for with an *action precision* parameter (α):

$$p(Action|\alpha) = \sigma(\alpha \times \ln p(Action|\pi)$$

Lower values of α increase the probability of selecting actions that disagree with beliefs about the optimal policy.

Based on our model, there are therefore several free parameters that could influence participant behavior: action precision (α), reward sensitivity (c_r), learning rate (η), and the starting value for concentration parameters at the beginning of each game (a_0; henceforth referred to as *insensitivity to information*). Lower values of α produce more randomness in behavior, which

could be associated with random exploration (primarily if on early trials). Lower values for c_r and a_0 produce greater directed exploration in different ways. Higher learning rates promote faster switches from exploration to exploitation. To arbitrate between different model choices, we estimated 10 different nested models – each with different choices in what model variables were included (or fixed at default values) and which to estimate. **Table 4** in the main text shows each model, as well as the default values used for each parameter if not estimated. Note that, based on our primary interest in goal-directed exploration vs. exploitation, c_r was always estimated. We then performed Bayesian model comparison (based on (Rigoux et al., 2014; Stephan et al., 2009)) to determine the best model.

For a tutorial introduction to this general modelling approach, see (Smith et al., 2022); for its implementation within our model, see the spm_MDP_VB_X MATLAB routine, freely available within the DEM (dynamic expectation maximization) toolbox of the most recent versions of SPM academic software (http://www.fil.ion.ucl.ac.uk/spm/). We have also included the modified version of this implementation for models assuming separate learning rates for wins and losses within our **Supplementary Code**. To illustrate the effect of the information value term in G_π, in **Supplementary Figure S1** we show example simulations comparing full model performance to a model where the information value term has been removed. Example simulations under different parameter settings are also shown in **Supplementary Figure S1**.

Parametric empirical Bayes (PEB) analysis details

The design matrix for PEB analyses (coded using sum coding for categorical variables) utilized columns for: age, sex (male = 1, female = -1), pre-morbid IQ (WRAT), group (HC = 1, SUD = -1), time (baseline = -1, follow-up = 1), and the interaction between group and time (HC/baseline = -1, HC/follow-up = 1, SUD/baseline = 1, SUD/follow-up = -1). Coding for all variables was detrended. For longitudinal analyses, columns for each subject were also included, and were coded using sum coding as well.

Further modeling diagnostics

In the main text, we provided results of parameter recoverability analyses in which synthetic data was generated from the winning (5 parameter) model under the range of parameter estimates found for our participants. Parameters were then estimated for this synthetic data, and it was shown that the generative and estimated parameters were strongly correlated, supporting recoverability. Here we report further supplementary diagnostic checks.

First, we assessed model identifiability within Bayesian model comparison. To do so, we generated simulated behavioral data from 131 synthetic participants (i.e., matching the number of study participants) using each of the 10 plausible models we considered. This simulated behavior was generated using the range of parameter values observed in our participants (and their default values for any parameters not included for the model in question). Each of the 10 models was then fit to each of the 10 sets of simulated data. Model comparison was then performed for each of the 10 sets of data to confirm whether the winning models corresponded to the models used to generate the data. Results are summarized in **Table S3**. As can be seen there, some but not all possible models were correctly identified. Of most importance, when the winning 5-parameter model (Model 9) generated the data, it was correctly identified in model comparison. However, Model 10 (the only other model that included separate learning rates for

wins and losses, but that did not include the "insensitivity to information" parameter) was also misidentified as Model 9. Thus, we cannot rule out the possibility that Model 10 offers a competitive account of participant behavior. Models with separate learning rates for wins and losses do appear identifiable more generally, however, which lends confidence to our results showing distinct effects of learning rates for losses.

Second, we assessed whether parameter estimates within the winning model showed any problematic dependence on choice of estimation priors. This is because variational methods can be vulnerable to local extrema within parameter space when assessing model fit, and we wished to confirm that results would not show meaningful differences under a different choice of starting parameter values. To assess this, we chose a different set of prior means and then re-estimated parameters under the winning model. We chose this alternative set of prior means to be equidistant and on the opposite side of (relative to our initial choice of prior means) the posterior group means in our primary results ($\alpha = 1.08$, $c_r = 5.26$, $\eta_{win} = 0.51$, $\eta_{loss} = 0.23$, $a_0 = 1.35$). Despite starting on the other side of the posterior means reported in the main text, we found that parameter estimates during this alternative round of estimation approached the same values found in our primary analyses (although they did not move all the way to those values due to the expected complexity cost). The correlations between posterior means under the original and alternate set of priors were as follows: α (r = 0.92, p < 0.001), c_r (r = 0.90, p < 0.001), η_{win} (r = 0.96, p < 0.001), η_{loss} (r = 0.96, p < 0.001), a_0 (r = 0.71, p < 0.001).

Correlations between model parameters and symptom severity at follow-up

Model parameters at follow-up did not show significant relationships with DAST scores at follow-up, although a suggestive trend was present for the action precision parameter: α (r = -.2, p = .09), c_r (r = -.17, p = .15), η_{win} (r = -.07, p = .52), η_{loss} (r = .18, p = .11), a_0 (r = -.12, p = .30). Relationships showed the same (non-significant) directional pattern when restricting to stimulant users or to opioid users, with notable trends in stimulant users for a_0 (r = -.25, p = .06) and α (r = -.24, p = .07).

Supplemental Tables and Figures

Table S1: Model-Free Task Measures in Full Dataset by Group and Session (Means and Standard Deviations) split by early (choices 2-7 per block) and late (choices 8-16) trials

	First/Second Half	Total	Baseline		Follow-up		Usable Data (N)	Effect of Clinical Status	Effect of Session	Effect of Clinical Status/Session Interaction
			HCs	SUDs	HCs	SUDs				
		131	48	83	48	83				
Wins	First Half	87.79 (7.39)	89.21 (6.75)	87.13 (7.61)	88.31 (6.97)	87.34 (7.75)	HC: 45<br>SUD+: 77<br>Total: 122	F(1, 117) = 2.83<br>p = 0.1<br>η2=0.02	F(1, 120) = 0.24<br>p = 0.62<br>η2=0	F(1, 120) = 0.08<br>p = 0.78<br>η2=0
	Second Half	93.31 (8.11)	93.62 (8.12)	91.61 (8.25)	93.96 (7.41)	94.45 (8.21)	HC: 45<br>SUD +: 77<br>Total: 122	F(1, 117) = 0.53<br>p = 0.47<br>η2=0	**F(1, 120) = 5.62**<br>**p = 0.02**<br>**η2=0.04**	F(1, 120) = 1.21<br>p = 0.27<br>η2=0.01

Win/Stay	First<br>Half	58.61<br>(17.46)	57.98<br>(18.2)	58.34<br>(17.67)	55.48<br>(17.76)	61.05<br>(16.59)	HC: 45<br>SUD +: 77<br>Total: 122	F(1, 117) = 0.32<br>p = 0.57<br>η2=0	F(1, 120) = 0.79<br>p = 0.38<br>η2=0.01	F(1, 120) = 1.54<br>p = 0.22<br>η2=0.01
	Second<br>Half	75.68<br>(18.75)	75.52<br>(17.41)	73.29<br>(20.55)	75.73<br>(18.15)	78.12<br>(17.93)	HC: 45<br>SUD +: 77<br>Total: 122	F(1, 117) = 0.19<br>p = 0.67<br>η2=0	**F(1, 120) = 4.02**<br>**p = 0.05**<br>**η2=0.03**	F(1, 120) = 1.7<br>p = 0.2<br>η2=0.01
Win/Shift	First<br>Half	17.56<br>(15.25)	19.46<br>(16.07)	17.25<br>(15.31)	21.17<br>(15.17)	14.7<br>(14.41)	HC: 45<br>SUD +: 77<br>Total: 122	F(1, 117) = 1.98<br>p = 0.16<br>η2=0.02	F(1, 120) = 1.77<br>p = 0.19<br>η2=0.01	F(1, 120) = 1.96<br>p = 0.16<br>η2=0.02
	Second<br>Half	17.52<br>(14.73)	18.42<br>(14.28)	18.2<br>(15.53)	17.9<br>(14.62)	16.1<br>(14.38)	HC: 45<br>SUD +: 77<br>Total: 122	F(1, 117) = 0.74<br>p = 0.39<br>η2=0.01	F(1, 120) = 1.78<br>p = 0.18<br>η2=0.01	F(1, 120) = 0.35<br>p = 0.55<br>η2=0
Lose/Stay	First<br>Half	20.69<br>(14.54)	16.54<br>(11.91)	21.05<br>(14.9)	18.12<br>(12.39)	24.2<br>(15.97)	HC: 45<br>SUD +: 77<br>Total: 122	**F(1, 117) = 9.83**<br>**p = 0.002**<br>**η2=0.08**	**F(1, 120) = 5.57**<br>**p = 0.02**<br>**η2=0.04**	F(1, 120) = 0.52<br>p = 0.47<br>η2=0
	Second<br>Half	26.43<br>(16.18)	22.79<br>(14.98)	25.37<br>(16.12)	27.85<br>(14.43)	28.76<br>(17.59)	HC: 45<br>SUD +: 77<br>Total: 122	**F(1, 117) = 4.43**<br>**p = 0.04**<br>**η2=0.04**	**F(1, 120) = 11.26**<br>**p = 0.001**<br>**η2=0.09**	F(1, 120) = 0.05<br>p = 0.82<br>η2=0
Lose/Shift	First<br>Half	43.14<br>(14.56)	46.02<br>(12.41)	43.36<br>(14.71)	45.23<br>(13.17)	40.05<br>(15.91)	HC: 45<br>SUD +: 77<br>Total: 122	**F(1, 117) = 5.66**<br>**p = 0.02**<br>**η2=0.05**	**F(1, 120) = 3.96**<br>**p = 0.05**<br>**η2=0.03**	F(1, 120) = 0.6<br>p = 0.44<br>η2=0
	Second<br>Half	40.38<br>(18.33)	43.27<br>(17.35)	43.13<br>(18.86)	38.52<br>(16.98)	37.02<br>(18.71)	HC: 45<br>SUD +: 77<br>Total: 122	F(1, 117) = 2.53<br>p = 0.11<br>η2=0.02	**F(1, 120) = 18.6**<br>**p < 0.001**<br>**η2=0.13**	F(1, 120) = 0.66<br>p = 0.42<br>η2=0.01

* Significant effects are bolded.

Table S2: Model-Free Task Measures in Matched Dataset by Group and Session (Means and Standard Deviations) split by early (choices 2-7 per block) and late (choices 8-16) trials

	First/Second Half	Total	Baseline		Follow-up		Usable Data (N)	Effect of Clinical Status	Effect of Session	Effect of Clinical Status/Session Interaction
			HCs	SUDs	HCs	SUDs				
		70	45	25	45	25				
Wins	First<br>Half	88.07<br>(7.29)	89.31<br>(6.96)	88.24<br>(6.02)	88.49<br>(6.81)	84.92<br>(9.15)	HC: 45<br>SUD+: 25<br>Total: 70	F(1, 65) = 3.6<br>p = 0.06<br>η2=0.05	F(1, 68) = 2.02<br>p = 0.16<br>η2=0.03	F(1, 68) = 0.98<br>p = 0.32<br>η2=0.01
	Second<br>Half	92.69<br>(7.76)	93.09<br>(8.09)	89.68<br>(6.82)	93.96<br>(7.48)	92.72<br>(8.21)	HC: 45<br>SUD +: 25<br>Total: 70	F(1, 65) = 1.89<br>p = 0.17<br>η2=0.03	F(1, 68) = 2.28<br>p = 0.14<br>η2=0.03	F(1, 68) = 0.92<br>p = 0.34<br>η2=0.01
Win/Stay	First<br>Half	58.13<br>(17.32)	58.2<br>(18.06)	58.56<br>(18.44)	56.96<br>(17.35)	59.68<br>(15.54)	HC: 45<br>SUD +: 25<br>Total: 70	F(1, 65) = 0.14<br>p = 0.71<br>η2=0	F(1, 68) = 0.02<br>p = 0.88<br>η2=0	F(1, 68) = 0.2<br>p = 0.66<br>η2=0
	Second<br>Half	74.31<br>(17.82)	74.76<br>(17.61)	71.08<br>(21.16)	75.29<br>(18.37)	75<br>(13.84)	HC: 45<br>SUD +: 25<br>Total: 70	F(1, 65) = 0.16<br>p = 0.69<br>η2=0	F(1, 68) = 0.56<br>p = 0.46<br>η2=0.01	F(1, 68) = 0.48<br>p = 0.49<br>η2=0.01
Win/Shift	First<br>Half	18.24<br>(15.1)	19.29<br>(15.73)	17.76<br>(16.57)	20<br>(14.79)	13.64<br>(12.69)	HC: 45<br>SUD +: 25<br>Total: 70	F(1, 65) = 1.56<br>p = 0.22<br>η2=0.02	F(1, 68) = 0.23<br>p = 0.64<br>η2=0	F(1, 68) = 1.17<br>p = 0.28<br>η2=0.02

	Second Half	18.54 (15.02)	18.82 (14.49)	19.28 (17.47)	18.09 (15.05)	18.12 (14.15)	HC: 45<br>SUD +: 25<br>Total: 70	F(1, 65) = 0<br>p = 0.95<br>η2=0	F(1, 68) = 0.23<br>p = 0.64<br>η2=0	F(1, 68) = 0.01<br>p = 0.91<br>η2=0
Lose/Stay	First Half	19.91 (14.54)	16.29 (12.13)	23.2 (15.34)	18.18 (12.4)	26.28 (18.8)	HC: 45<br>SUD +: 25<br>Total: 70	**F(1, 65) = 6.77**<br>**p = 0.01**<br>**η2=0.09**	F(1, 68) = 1.82<br>p = 0.18<br>η2=0.03	F(1, 68) = 0.11<br>p = 0.74<br>η2=0
	Second Half	26.28 (16.11)	22.02 (15.1)	27.8 (15.37)	27.04 (14.43)	31.04 (20.13)	HC: 45<br>SUD +: 25<br>Total: 70	F(1, 65) = 2.44<br>p = 0.12<br>η2=0.04	**F(1, 68) = 5.69**<br>**p = 0.02**<br>**η2=0.08**	F(1, 68) = 0.22<br>p = 0.64<br>η2=0
Lose/Shift	First Half	43.72 (14.15)	46.22 (12.71)	40.48 (14.91)	44.87 (13.41)	40.4 (16.57)	HC: 45<br>SUD +: 25<br>Total: 70	F(1, 65) = 3.53<br>p = 0.06<br>η2=0.05	F(1, 68) = 0.22<br>p = 0.64<br>η2=0	F(1, 68) = 0.1<br>p = 0.75<br>η2=0
	Second Half	40.86 (17.63)	44.4 (17.3)	41.84 (18.09)	39.58 (16.93)	35.84 (18.58)	HC: 45<br>SUD +: 25<br>Total: 70	F(1, 65) = 0.95<br>p = 0.33<br>η2=0.01	**F(1, 68) = 7**<br>**p = 0.01**<br>**η2=0.09**	F(1, 68) = 0.08<br>p = 0.78<br>η2=0

* Significant effects are bolded.

Table S3: Results of Model Identifiability Analyses during Model Comparison

	α	c_r	η	a_0	**Model Comparison Results**
**Parameter:**	**action precision**	**reward sensitivity**	**learning rate**	**insensitivity to information**	
**Default value if not estimated**	4	always estimated	removed from model	0.25	
**Prior means during estimation***	4	4	0.5	0.25	
**Model 1**	**Y**	**Y**	N	N	**M8: *pxp* = 1**
**Model 2**	**Y**	**Y**	**Y**	N	**M3: *pxp* = 1**
**Model 3**	**Y**	**Y**	**Y**	**Y**	**M3: *pxp* = 1***
**Model 4**	N	**Y**	**Y**	**Y**	**M3: *pxp* = 1**
**Model 5**	N	**Y**	**Y**	N	M3: *pxp* = .19<br>**M4: *pxp* = .81**
**Model 6**	N	**Y**	N	N	**M6: *pxp* = 1***
**Model 7**	N	**Y**	N	**Y**	**M3: *pxp* = 1**
**Model 8**	**Y**	**Y**	N	**Y**	**M3: *pxp* = 1**
**Model 9****	**Y**	**Y**	Wins/Losses	**Y**	**M9: *pxp* = 1***
**Model 10**	**Y**	**Y**	Wins/Losses	N	**M9: *pxp* = 1**

Note: *pxp* = protected exceedance probability. *Indicates that the generative model was correctly identified. **Indicates the winning model used within further analyses in the main text.

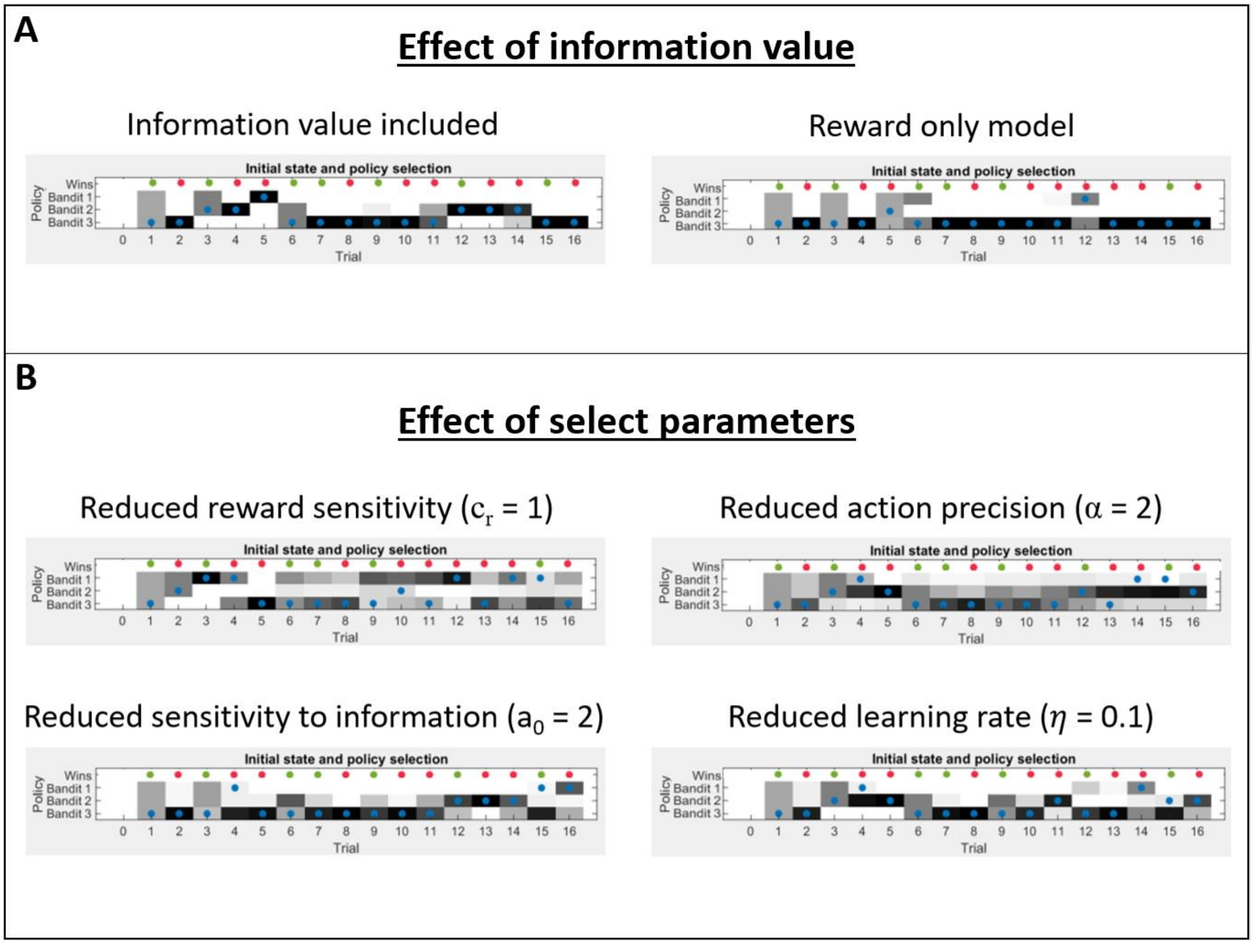

Figure S1. (A) Example model simulation of one game with and without the information value term of the expected free energy included in policy valuation. Reward probabilities for bandits 1-3 in this game are 0.46, 0.49, and .64 (respectively). Darker shades indicate higher choice probabilities; blue circles indicate the action taken; red and green circles indicate losses and wins (respectively). While the agent on the left panel is driven by both reward maximization and information gain, the agent on the right panel only cares about reward. This induces subtle differences in predictions for behavior that are visible, for example, at time step three. Here, after having observed one rewarding and one non-rewarding outcome in bandit three, the agent on the left now prefers to gain information about the other two bandits, whereas the agent on the right equally prefers the three bandits because they all have a reward value of 0.5. Here, action precision ($\boldsymbol{\alpha}$; lower values promoting random exploration) was set to a high value of 16 to highlight the effects of goal-directed exploration. Reward sensitivity ($\boldsymbol{c_r}$; lower values promote goal-directed exploration) was set to 4, learning rates were set to 0.5, and the prior concentration parameters in the observation model, governing insensitivity to information, were defined as 0.25. **(B)** Example model simulations under single changes from the above-stated parameter values. As can be seen here**,** reduced $\boldsymbol{c_r}$ leads to over-exploration, while reduced sensitivity to information leads to behavior similar to the reward-only model. Reduced $\boldsymbol{\alpha}$ leads to more stochastic behavior, and reduced learning rate leads to less confident choices in later trials.

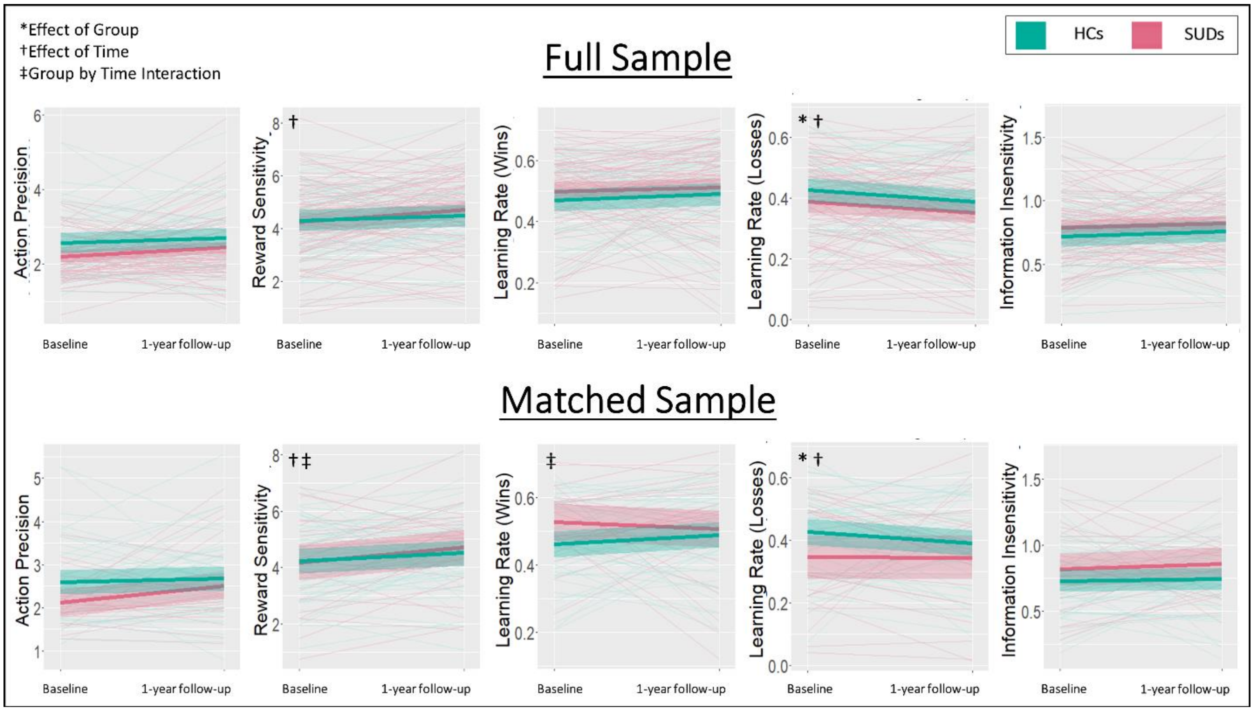

Figure S2. Spaghetti plots showing individual changes from baseline to follow-up, as well as group means and standard errors, for all model parameters in the full and matched samples.

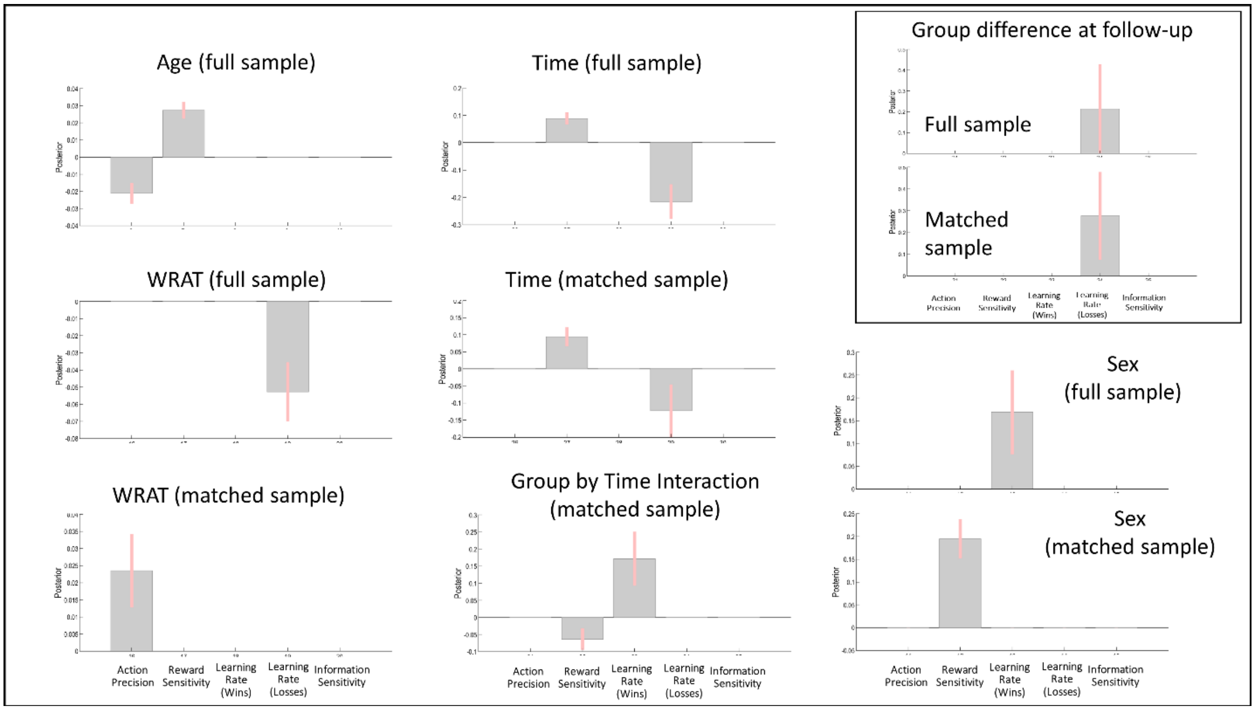

Figure S3. Illustration of effect sizes for additional effects found in PEB analyses that were not illustrated in the main text. For the group by time interaction, a positive value indicates increases over time in HCs and decreases over time in SUDs.

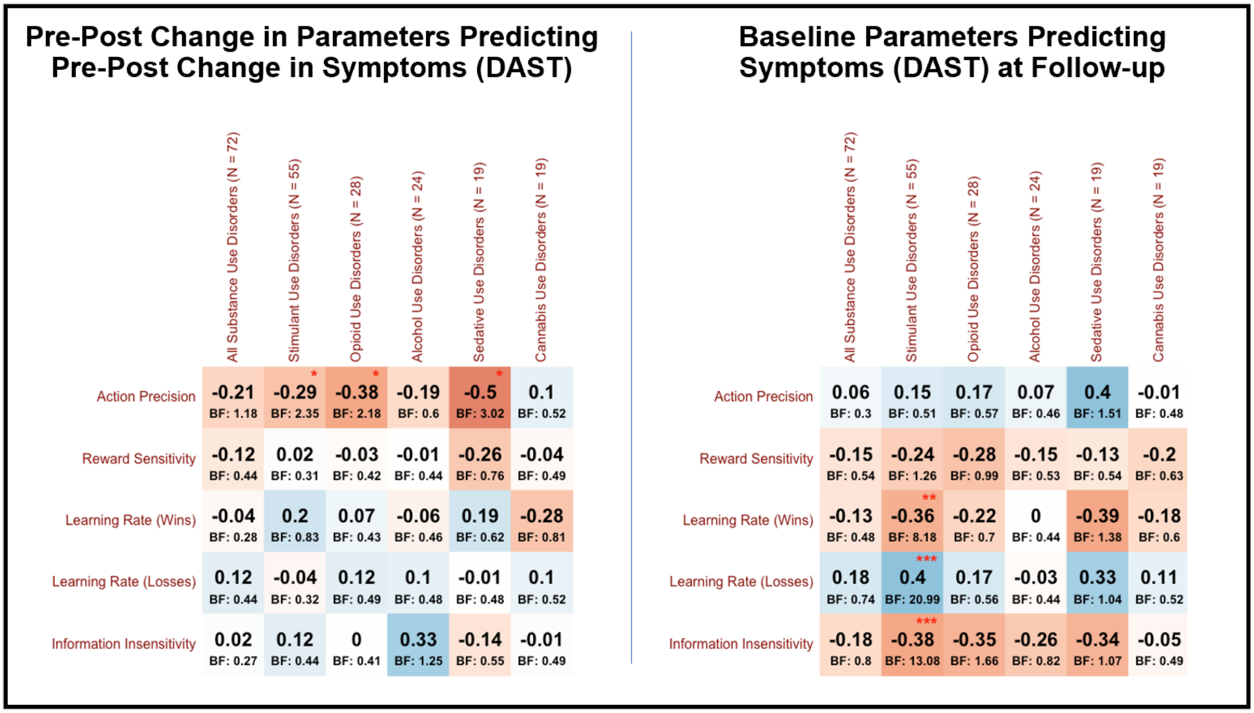

Figure S4. *Left*: Correlation between pre-to-post changes in model parameters and pre-to-post changes in symptom severity (DAST) in the full SUD sample and in subsamples restricted to only individuals meeting criteria for specific SUDs. DAST change scores account for what could already be predicted based on age, sex, and premorbid IQ. *Right*: Predictive relationships between baseline model parameters and symptom severity (DAST) at 1-year follow-up in the full SUD sample and in subsamples restricted to only individuals meeting criteria for specific SUDs. DAST scores account for what could already be predicted based on age, sex, and premorbid IQ. BF indicates the Bayes factor for each correlation. *p < .05, **p < .01, ***p < .001 (uncorrected).

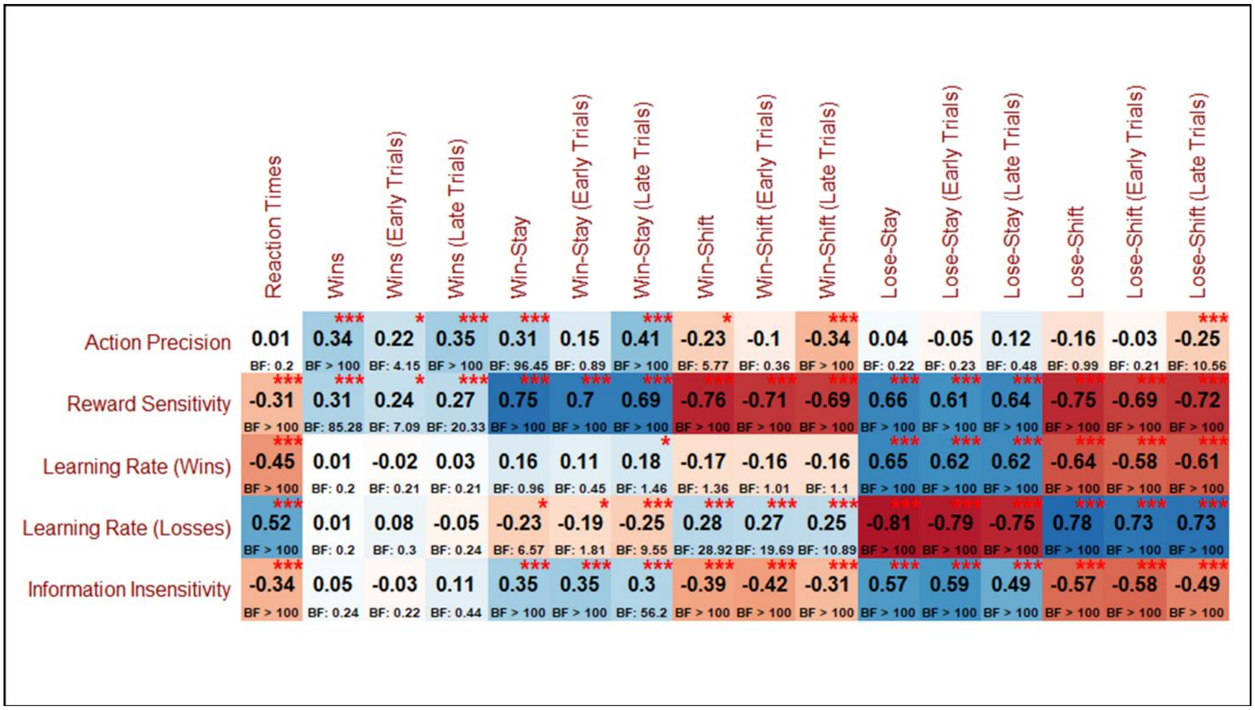

Figure S5. Correlations between model parameters and model-free behavior. Early trials = trials 2-7 per game. Late trials = trials 8-15 per game. BF indicates the Bayes factor for each correlation. **p* < .05, ***p* < .01, ****p* < .001 (uncorrected).

References

Da Costa, L., Parr, T., Sajid, N., Veselic, S., Neacsu, V., Friston, K., 2020. Active inference on discrete state-spaces: A synthesis. J Math Psychol 99, 102447.

Friston, K., FitzGerald, T., Rigoli, F., Schwartenbeck, P., Pezzulo, G., 2017. Active Inference: A Process Theory. Neural Comput 29(1), 1-49.

Friston, K.J., Parr, T., de Vries, B., 2017. The graphical brain: Belief propagation and active inference. Netw Neurosci 1(4), 381-414.

Parr, T., Friston, K.J., 2017. Working memory, attention, and salience in active inference. Sci Rep 7(1), 14678.

Rigoux, L., Stephan, K.E., Friston, K.J., Daunizeau, J., 2014. Bayesian model selection for group studies - revisited. Neuroimage 84, 971-985.

Smith, R., Friston, K.J., Whyte, C.J., 2022. A step-by-step tutorial on active inference and its application to empirical data. Journal of Mathematical Psychology 107, 102632.

Stephan, K.E., Penny, W.D., Daunizeau, J., Moran, R.J., Friston, K.J., 2009. Bayesian model selection for group studies. Neuroimage 46(4), 1004-1017.
